# Comparison of individual-level and population-level risk factors for rhinoconjunctivitis, asthma, and eczema in the International Study of Asthma and Allergies in Childhood (ISAAC) Phase Three

**DOI:** 10.1016/j.waojou.2020.100123

**Published:** 2020-07-02

**Authors:** Charlotte E. Rutter, Richard J. Silverwood, M.Innes Asher, Philippa Ellwood, Neil Pearce, Luis Garcia-Marcos, David P. Strachan, the ISAAC Phase Three Study Group

**Affiliations:** a Department of Medical Statistics, London School of Hygiene and Tropical Medicine, London, United Kingdom; b Centre for Longitudinal Studies, Department of Social Science, University College London, London, United Kingdom; c Department of Paediatrics: Child and Youth Health, Faculty of Medical and Health Sciences, University of Auckland, New Zealand; d Centre for Global NCDs, London School of Hygiene and Tropical Medicine, London, United Kingdom; e Pediatric Allergy and Pulmonology Units, ‘Virgen de La Arrixaca’ University Children's Hospital, University of Murcia, ARADyAL Network and IMIB Bioresearch Institute, Murcia, Spain; f Population Health Research Institute, St George's University of London, London, United Kingdom

**Keywords:** Rhinoconjunctivitis, Asthma, Eczema, Multimorbidity, Global

## Abstract

**Background:**

Symptoms of asthma, allergic rhinoconjunctivitis, and atopic eczema in children cluster at both the individual and population levels.

**Objectives:**

To assess individual-level and school-level risk factors for symptoms of rhinoconjunctivitis and compare them to corresponding associations with symptoms of asthma and eczema in Phase Three of the International Study of Asthma and Allergies in Childhood.

**Methods:**

We studied 116,863 children aged 6–7 years from 2163 schools in 59 centres and 22 countries and 224,436 adolescents aged 13–14 years from 2037 schools in 97 centres in 41 countries. Multilevel logistic regression models were fitted with random intercepts for school, centre, and country, adjusting for sex and maternal education at the child level. Associations between symptoms and a range of lifestyle and environmental risk factors were assessed for both the child's exposure and mean exposure at the school. Models were fitted for rhinoconjunctivitis, asthma, and eczema singly (unimorbidity) and for combinations of these conditions (multimorbidity).

**Results:**

Generally, associations between symptoms and exposures at the school level were similar in direction and magnitude to those at the child level. Associations with multimorbidity were stronger than for unimorbidity, particularly in individuals with symptoms of all three diseases, but risk factor associations found in conventional single disease analyses persisted among children with only one condition, after excluding multimorbid groups.

Comparisons of individuals with only one disease showed that many risk factor associations were consistent across the three conditions. More strongly associated with asthma were low birthweight, cat exposure in infancy, and current maternal smoking. Current paracetamol use was more strongly associated with asthma and rhinoconjunctivitis than eczema. Breastfeeding was more strongly associated with eczema than asthma or rhinoconjunctivitis.

The direction and magnitude of most risk factor associations were similar in affluent and non-affluent countries, although notable exceptions include farm animal contact in infancy and larger sibships, which were associated with increased risk of rhinoconjunctivitis in non-affluent countries but reduced risk in affluent countries. In both age groups, current paracetamol use increased risk of each disease to a greater extent in affluent countries than in non-affluent countries. Effects of paracetamol and antibiotics in infancy were more consistent between richer and poorer settings.

**Conclusions:**

Most of the environmental and lifestyle correlates of rhinoconjunctivitis, asthma and eczema in childhood display similarity across the three conditions, even in less affluent settings where allergic sensitisation is less likely to explain the concordant epidemiological patterns.

**Trial registration:**

Not applicable.

## Introduction

The International Study of Asthma and Allergies in Childhood (ISAAC) has used standardised questionnaires to assess prevalence, time trends, and epidemiological associations for symptoms of non-infective rhinoconjunctivitis, asthma, and eczema among children from over 300 centres in more than 100 countries worldwide.^1^^,^^2^ More detailed biomedical assessment in 30 diverse centres in ISAAC Phase Two^3^, ^4^, ^5^ has demonstrated that allergic sensitisation accounts for a much lower proportion of rhinoconjunctivitis, asthma, and eczema symptoms in centres from low- and middle-income countries than it does in more affluent settings which feature more prominently in the epidemiological literature.

Previous publications from ISAAC Phase Three have presented the associations of each of the 3 diseases with single environmental or lifestyle factors.^6^, ^7^, ^8^, ^9^, ^10^, ^11^, ^12^, ^13^, ^14^, ^15^, ^16^, ^17^, ^18^, ^19^ More recently, these have been summarised across multiple risk factors for symptoms of asthma^20^ and eczema,^21^ and comparisons have been made between the relationship of each of these diseases to exposures measured at the level of individuals and exposures averaged at the area level (schools).

In this paper, we apply the multi-level analytical approach to symptoms of rhinoconjunctivitis and extend our overview to assess similarities and differences in the epidemiological patterns of the 3 diseases, singly and in combination. We also compare these patterns between centres from higher-income and lower-income countries.

## Methods

### Study design

A brief summary of the ISAAC Phase Three methods are presented in this paper and more details are available elsewhere.^1^ ISAAC Phase Three was a multi-centre, multi-country, cross-sectional study of children (age 6–7 years) and adolescents (age 13–14 years). Within a defined geographical area (centre), a sample of schools were chosen at random. All children within the age groups in those schools were asked to participate.^1^ The Phase Three survey took place in 2000–2003 and included two standardised questionnaires (http://isaac.auckland.ac.nz); the original symptom questionnaire from ISAAC Phase One^1^^,^^2^ with information on symptoms of asthma, eczema and rhinoconjunctivitis, and an environmental questionnaire which collected data on a range of possible risk factors for the development of these disorders.^1^

### Variable definitions

The 3 main outcomes of interest, asthma, eczema, and rhinoconjunctivitis, are defined using previous ISAAC conventions.^1^, ^2^, ^3^ These 3 diseases are likely to be undiagnosed in many cases as people seek to self-treat (particularly rhinoconjunctivitis), and the rate of doctor diagnoses is likely to vary widely from country to country. Thus, outcomes assessed in the ISAAC questionnaire are based on a description of symptoms rather than a diagnosis of disease.

Rhinoconjunctivitis is defined by positive responses to all of the following 3 questions:

*Have you [has your child] ever had a problem with sneezing, or a runny, or blocked nose when you did not have a cold or the flu?*

*In the past 12 months, have you [has your child] had a problem with sneezing, or a runny, or blocked nose when you [he/she] did not have a cold or the flu?*

*In the past 12 months, has this nose problem been accompanied by itchy-watery eyes?*

Eczema is defined by positive responses to all of the following 3 questions:

*Have you [has your child] ever had an itchy rash which was coming and going for at least six months?*

*Have you [has your child] had this itchy rash at any time in the past 12 months?*

*Has this itchy rash at any time affected any of the following places: the folds of the elbows, behind the knees, in front of the ankles, under the buttocks, or around the neck, ears or eyes?*

Asthma is defined by a positive response to the following question:

*Have you [has your child] had wheezing or whistling in the chest in the past 12 months?*

The environmental questionnaire for the 6-7-year-old age group contained more questions on early life exposures as this was completed by the parents of the child. We restricted our analyses to the risk factors which had shown associations with either rhinoconjunctivitis, asthma, or eczema symptoms in the last 12 months in previous analyses at the individual level. Variables included for the younger age group (6–7 years) were paracetamol use in the first year of life and in the past 12 months,^6^ antibiotic use in the first year of life,^7^ breast feeding,^8^ pets in the home in the first year of life,^9^ regular contact with farm animals in the first year of life, and prenatally through maternal contact,^10^ truck traffic in the last 12 months,^11^ fast food consumption in the last 12 months,^12^ television viewing in the last 12 months,^13^ parental smoking in the last 12 months,^14^ open fire cooking,^15^ birth weight,^16^ and number of siblings.^17^ For the older age group (13–14 years), truck traffic,^11^ fast food consumption,^12^ television viewing,^13^ parental smoking,^14^ and paracetamol use,^18^ all in the past 12 months, open fire cooking^15^ and number of siblings^17^ were included.

Most of the above risk factors were parameterised as binary variables from “yes/no” questions in the environmental questionnaire. The exceptions were: paracetamol use in the past 12 months (at least once per month vs. less than once per month), heavy truck traffic (frequently or almost the whole day vs. seldom or never), fast food consumption (once per week or more vs. less than once per week), television viewing (at least 1 h per day vs. less than 1 h per day), birth weight (less than 2.5 kg vs. at least 2.5 kg), and number of siblings (2 or more siblings vs. 1 or no siblings). Full definitions are in Table S1, Supporting Material. The highest level of maternal education was recorded as primary, secondary, tertiary or missing/not stated.

Gross National Income (GNI) in 2002 was obtained from the World Bank website^22^ where available, with gaps filled by the CIA World Factbook.^23^ Countries were classified as ‘affluent’ or ‘non-affluent’ using a 2001 GNI value of US$9205 per capita as a cut-off, which separates high-income countries from low and middle-income countries.^24^

### Statistical analyses

Separate analyses were conducted for the 2 age groups. Centres with fewer than 1000 individuals in an age group were excluded from the analyses for that age group. Each school was required to have at least 10 individuals to be included in the analyses for that age group. In addition, a response rate of at least 60% was required for children and at least 70% for adolescents for a centre to be included.

Mixed effect (multilevel) logistic regression models were used for all analyses with random intercepts at the 3 highest levels of the four-level hierarchy: individuals, schools, centres and countries (from lowest to highest). All analyses additionally adjusted for sex and maternal education as confounders at the individual level.

The potential risk factors for rhinoconjunctivitis symptoms were compared at individual level and at school level in a similar way to previous publications on asthma^20^ and eczema.^21^ The school-level risk factors are less prone to reverse causation bias than the individual-level risk factors as a change in behaviour of a few people with the disease will not greatly affect the school-level prevalence of that risk factor. Thus, similar results at both levels can be interpreted as suggestive evidence against reverse causation influencing individual-level associations.

For comparison of risk factor associations between the 3 different diseases, 3 different modelling approaches were used:

i) Standard outcomes - modelling the 3 disease outcomes separately but within the same sample of children,
ii) Multimorbid outcomes - modelling each of the different combination of disease outcomes (i.e. asthma only, eczema only, rhinoconjunctivitis only, asthma and eczema, asthma and rhinoconjunctivitis, eczema and rhinoconjunctivitis, and all 3) against those with no disease and comparing the resulting risk factor associations, and
iii) Unimorbid outcomes - comparing individuals with only asthma, only eczema or only rhinoconjunctivitis symptoms in the last 12 months and modelling the 3 combinations of disease pairs to evaluate if the risk factors are more associated with one disease than another.

In each of these modelling analyses we checked for collinearity between the risk factors by comparing the standard errors in the fully adjusted model (all risk factors and confounders) to those in minimally adjusted models (only the risk factor of interest and the confounders).

Additionally, we ran each model separately for "affluent" and "non-affluent" countries (with the exception of the multimorbid outcomes analyses where some of the sample sizes were too small). We also tested for an interaction between country affluence and each risk factor individually. Analyses were conducted using Stata version 15.^25^

## Results

### Derivation and characteristics of the sample analysed

In the age 6–7 analyses there were 75 centres (comprising 221,280 children) that met the standard ISAAC inclusion criteria^1^ of a minimum of 1000 children and a response rate of at least 60%. For multi-level analysis, 263 schools (1427 children in total) were excluded due to having fewer than 10 children and a further 102,990 children excluded for not having data available for all 3 outcomes (asthma, eczema, and rhinoconjunctivitis symptoms), confounders (sex and mother's level of education), and all the included risk factors. The remaining 116,863 children, on which these results are based (the “synthesis sample”), were from 2163 schools within 59 centres, in 22 different countries (Fig. S1, Supporting Material).

The prevalence of rhinoconjunctivitis symptoms among the 6-7-year-olds included in this analysis was 8.9%, asthma symptoms was 9.7%, and eczema symptoms was 7.3%. The overall prevalence of the exposures ranged from 1.8% for current open fire cooking to 80.5% for ever breastfed. These and further summary statistics for the synthesis sample are presented in Table 1.

**Table 1:** Summary statistics for variables and their prevalence in subjects who had data present for the 3 outcomes, the confounders sex and maternal education level and all other exposures of interest in the table (the “synthesis sample”)

		Age 6–7 years (n = 116,863)						Age 13–14 years (n = 224,436)					
		Individual-level prevalence	Centre-level prevalence (%) quartiles					Individual-level Prevalence	Centre-level prevalence (%) quartiles				
Type	Variable	(%)	Min	Q1	Med	Q3	Max	(%)	Min	Q1	Med	Q3	Max
Outcome	Rhinoconjunctivitis in the past 12 months	8.9	0.9	3.7	7.5	12.2	25.2	14.1	1.2	8.8	13.2	18.5	31.7
	Asthma symptoms in the past 12 months	9.7	2.5	5.4	9.0	13.2	29.7	10.6	0.7	6.1	9.8	14.4	32.4
	Eczema symptoms in the past 12 months	7.3	0.6	2.5	6.0	10.9	18.9	6.2	0.1	3.0	4.7	8.0	23.8
Multiple													
outcome	No symptoms	80.1	62.2	74.1	80.1	89.0	95.0	76.0	51.8	69.6	75.8	83.5	98.2
	Rhinoconjunctivitis only	4.7	0.3	2.2	3.6	5.2	16.5	8.8	1.1	5.0	8.4	11.1	22.4
	Asthma only	5.8	1.7	3.6	5.0	7.4	26.1	6.1	0.6	3.7	5.5	8.0	19.6
	Eczema only	4.4	0.6	1.8	3.2	5.5	11.4	3.2	0.0	1.6	2.5	4.1	13.4
	Rhinoconjunctivitis and Asthma	2.1	0.2	0.8	2.0	3.3	4.9	2.9	0.0	1.4	2.6	4.0	10.3
	Rhinoconjunctivitis and Eczema	1.1	0.0	0.3	0.8	1.6	4.3	1.4	0.0	0.5	0.9	1.6	8.0
	Asthma and Eczema	0.9	0.0	0.3	0.6	1.3	3.9	0.7	0.0	0.2	0.5	1.0	2.9
	Symptoms of all three	0.9	0.0	0.2	0.8	1.4	3.3	0.9	0.0	0.3	0.8	1.2	4.1
Early life													
exposure	Low birthweight	7.7	0.0	5.2	6.2	9.4	39.4	NA	NA	NA	NA	NA	NA
	Breastfed ever	80.5	29.2	79.3	84.7	91.2	97.0	NA	NA	NA	NA	NA	NA
	Farm animals (prenatal)	7.7	0.7	4.5	7.5	10.7	24.2	NA	NA	NA	NA	NA	NA
	Farm animals (1st year)	9.3	1.9	6.2	9.3	13.5	24.9	NA	NA	NA	NA	NA	NA
	Cat (1st year)	10.9	1.2	4.9	8.3	11.9	53.8	NA	NA	NA	NA	NA	NA
	Dog (1st year)	19.8	0.7	10.6	18.2	27.6	46.3	NA	NA	NA	NA	NA	NA
	Paracetamol (1st year)	66.1	8.6	59.0	68.1	82.7	93.9	NA	NA	NA	NA	NA	NA
	Antibiotics (1st year)	55.6	18.8	52.0	57.8	62.3	77.7	NA	NA	NA	NA	NA	NA
Current													
exposure	2 or more siblings	34.7	12.4	21.9	31.8	45.3	83.1	53.9	3.9	37.9	57.6	73.3	100.0
	Heavy truck traffic (past 12 months)	37.9	6.3	32.1	37.8	43.8	67.4	39.5	15.2	32.3	38.0	45.0	90.9
	Fast food (past 12 months)	39.6	9.3	20.4	42.4	54.3	98.1	53.6	6.1	42.7	53.7	66.4	98.3
	Television (past 12 months)	80.1	40.1	72.9	82.2	89.1	95.3	85.7	42.9	80.9	90.0	93.2	98.0
	Paternal tobacco (past 12 months)	31.7	3.2	19.9	28.9	43.6	55.3	38.5	2.7	23.9	36.4	46.4	94.1
	Maternal tobacco (past 12 months)	16.3	0.0	1.6	12.6	24.5	46.6	18.3	0.4	2.7	14.1	28.9	93.7
	Paracetamol (past 12 months)	17.8	0.0	9.9	15.7	23.9	65.3	26.7	0.0	18.4	28.6	34.7	66.0
	Open fire cooking	1.8	0.0	0.3	1.1	2.0	44.8	5.2	0.0	0.6	1.3	4.1	86.1

For the 13-14 year-olds there were 122 centres (comprising 362,048 adolescents) meeting the ISAAC criteria^1^ of a minimum of 1000 per centre and a response rate of at least 70%. For multi-level analysis, 64 schools (comprising 298 individuals) were excluded due to having fewer than 10 adolescents. A further 137,314 individuals were excluded for not having data available for all three outcomes (asthma, eczema and rhinoconjunctivitis symptoms), confounders (sex and mother's level of education) and all the included risk factors of interest. The remaining “synthesis sample” contained 224,436 adolescents from 2037 schools within 97 centres, in 41 different countries (Fig. S2, Supporting Material).

The prevalence of rhinoconjunctivitis symptoms among the 13-14-year-olds included in this analysis was 14.1%, asthma symptoms was 10.6%, and eczema symptoms was 6.2%. The overall prevalence of the exposures ranged from 5.2% for current open fire cooking to 85.7% for watching television at least an hour a day. For further details, see Table 1.

### Multi-level models for rhinoconjunctivitis

Table 2 presents associations at the individual level (within schools) and the area level (between schools, within centre) for exposures of interest, adjusted for sex and mother's educational level (“minimally adjusted”), and for each other (“fully adjusted”), as derived from the multi-level model.

**Table 2:** Individual-level (within school) and school-level (between school) effects of exposures on rhinoconjunctivitis symptoms using the synthesis sample^a^. Mixed logistic regression models with random intercepts at the school, centre and country levels.

Age group	Exposures of interest	Minimally adjusted model^b^		Fully adjusted model^c^	
		Individual-level	School-level	Individual-level	School-level
		OR (95% CI)	OR (95% CI)	OR (95% CI)	OR (95% CI)
6–7 years (n = 116,863)	Low birthweight	1.11 (1.02, 1.20)	3.03 (1.88, 4.88)	1.04 (0.96, 1.13)	2.59 (1.56, 4.29)
	Breastfed ever	0.97 (0.92, 1.02)	0.52 (0.37, 0.73)	1.00 (0.95, 1.06)	0.62 (0.44, 0.88)
	Farm animals (prenatal)	1.36 (1.27, 1.46)	1.56 (1.10, 2.22)	1.18 (1.07, 1.30)	1.16 (0.61, 2.20)
	Farm animals (1st year)	1.30 (1.21, 1.39)	1.55 (1.11, 2.16)	1.07 (0.98, 1.17)	1.19 (0.65, 2.18)
	Cat (1st year)	1.19 (1.11, 1.27)	1.36 (0.94, 1.97)	1.09 (1.01, 1.16)	1.10 (0.72, 1.68)
	Dog (1st year)	1.16 (1.10, 1.21)	1.21 (0.89, 1.63)	1.07 (1.01, 1.12)	0.94 (0.67, 1.31)
	Paracetamol (1st year)	1.80 (1.71, 1.89)	1.32 (1.01, 1.75)	1.39 (1.32, 1.47)	0.99 (0.73, 1.35)
	Antibiotics (1st year)	1.84 (1.75, 1.92)	1.45 (1.09, 1.91)	1.58 (1.51, 1.66)	1.39 (1.03, 1.88)
	2 or more siblings	0.99 (0.95, 1.04)	1.04 (0.83, 1.30)	0.98 (0.94, 1.03)	0.88 (0.69, 1.12)
	Heavy truck traffic (current)	1.25 (1.20, 1.31)	1.12 (0.90, 1.40)	1.17 (1.12, 1.22)	0.92 (0.73, 1.16)
	Fast food (current)	1.04 (0.99, 1.09)	1.13 (0.89, 1.42)	0.99 (0.94, 1.03)	1.04 (0.82, 1.32)
	Television (current)	0.98 (0.92, 1.03)	1.59 (1.18, 2.14)	0.93 (0.88, 0.98)	1.45 (1.05, 2.01)
	Paternal tobacco (current)	1.11 (1.06, 1.16)	1.34 (1.03, 1.74)	1.07 (1.02, 1.12)	0.86 (0.63, 1.19)
	Maternal tobacco (current)	1.12 (1.06, 1.19)	1.67 (1.25, 2.23)	1.05 (0.99, 1.11)	1.39 (0.98, 1.96)
	Paracetamol (current)	2.30 (2.18, 2.42)	2.30 (1.66, 3.20)	2.02 (1.92, 2.13)	2.04 (1.43, 2.89)
	Open fire cooking (current)	1.03 (0.86, 1.23)	2.21 (1.16, 4.22)	0.99 (0.82, 1.19)	1.85 (0.92, 3.71)
13–14 years (n = 224,436)	2 or more siblings	1.05 (1.02, 1.08)	1.02 (0.83, 1.25)	1.04 (1.01, 1.07)	0.93 (0.75, 1.15)
	Heavy truck traffic (current)	1.27 (1.24, 1.30)	1.35 (1.09, 1.67)	1.23 (1.20, 1.26)	1.16 (0.94, 1.44)
	Fast food (current)	1.10 (1.07, 1.13)	1.31 (1.07, 1.61)	1.06 (1.03, 1.08)	1.24 (1.02, 1.51)
	Television (current)	1.04 (1.00, 1.08)	1.35 (0.97, 1.88)	1.01 (0.97, 1.05)	1.20 (0.86, 1.68)
	Paternal tobacco (current)	1.16 (1.13, 1.19)	1.08 (0.84, 1.40)	1.10 (1.07, 1.13)	0.75 (0.55, 1.00)
	Maternal tobacco (current)	1.21 (1.17, 1.25)	1.46 (1.08, 1.97)	1.14 (1.10, 1.17)	1.56 (1.12, 2.18)
	Paracetamol (current)	1.80 (1.75, 1.85)	3.52 (2.69, 4.60)	1.76 (1.71, 1.81)	3.48 (2.66, 4.56)
	Open fire cooking (current)	1.16 (1.08, 1.25)	1.73 (1.19, 2.49)	1.16 (1.08, 1.26)	2.02 (1.39, 2.93)

a Synthesis sample contains individuals with data present for all 3 outcomes, sex, maternal education and all exposures of interest.

b Adjusted for sex and mothers level of education.

c Adjusted for sex, mothers level of education and all other variables in the table (within age group)

For the 6–7 age group, the strongest mutually adjusted associations with rhinoconjunctivitis at the individual level were current paracetamol use (odds ratio = 2.02; 95% CI = 1.92–2.13) and antibiotics in the first year (1.58; 1.51–1.66). These associations were very similar at the school level with odds ratios 2.04 (1.43–2.89) and 1.39 (1.03–1.88) respectively. However, the weaker child-level association with early paracetamol use (1.39; 1.32–1.47) was not seen at the school level (0.99; 0.73–1.35). Similarly, a modest child-level association with heavy truck traffic (1.17; 1.12–1.22) was inconsistent with the inverse relationship at school level (odds ratio 0.92; 0.73–1.16). Low birthweight showed no evidence of an association with rhinoconjunctivitis at the individual level (1.04; 0.96–1.13), but at the school level the association was strong and significant (2.59; 1.56–4.29). Similarly, a school-level association was evident for television viewing (1.45; 1.05–2.01) but not for children within schools (0.93; 0.88–0.98).

In the 13–14 age group, there was a strong association at the individual level with current paracetamol use (1.76; 1.71–1.81) which was even stronger at the school-level (3.48; 2.66–4.56). A less strong child-level association was observed for heavy truck traffic (1.23; 1.20–1.26), which was consistent at the school level (1.16; 0.94–1.44) though there was less precision on the latter estimate. Paternal and maternal smoking had similar effects at the individual-level, but at the school level their associations were in opposite directions. For cooking by open fire, the school-level association (2.02; 1.39–2.93) was much stronger than the child-level association (1.16; 1.08–1.26).

The precision of estimates from the fully adjusted models and those from the corresponding minimally adjusted models (Table 2) were compared and no evidence of collinearity was found.

### Comparison of risk factor patterns for rhinoconjunctivitis, asthma and eczema

Table 3 compares, by age group, the individual-level associations of each exposure with symptoms of rhinoconjunctivitis (from Table 2), asthma, and eczema (previously published in slightly different samples^20^^,^^21^ but reanalysed here on the same “synthesis sample” as for rhinoconjunctivitis).

**Table 3:** Single outcome models of fully adjusted^a^, individual-level (within school) effects of exposures using the synthesis sample^b^. Mixed logistic regression models with random intercepts at the school, centre and country levels.

Age group	Exposures of interest	Fully adjusted model^a^		
		Rhinoconjunctivitis symptoms	Asthma symptoms	Eczema symptoms
		OR (95% CI)	OR (95% CI)	OR (95% CI)
6–7 years (n = 116,863)	Low birthweight	1.04 (0.96, 1.13)	1.15 (1.07, 1.25)	0.90 (0.82, 0.99)
	Breastfed ever	1.00 (0.95, 1.06)	0.95 (0.90, 1.00)	1.11 (1.04, 1.17)
	Farm animals (prenatal)	1.18 (1.07, 1.30)	1.19 (1.08, 1.30)	1.12 (1.01, 1.24)
	Farm animals (1st year)	1.07 (0.98, 1.17)	0.98 (0.89, 1.06)	1.13 (1.03, 1.25)
	Cat (1st year)	1.09 (1.01, 1.16)	1.22 (1.14, 1.30)	1.10 (1.02, 1.18)
	Dog (1st year)	1.07 (1.01, 1.12)	1.03 (0.98, 1.08)	1.06 (1.00, 1.12)
	Paracetamol (1st year)	1.39 (1.32, 1.47)	1.34 (1.27, 1.41)	1.29 (1.22, 1.37)
	Antibiotics (1st year)	1.58 (1.51, 1.66)	1.66 (1.59, 1.74)	1.40 (1.33, 1.47)
	2 or more siblings	0.98 (0.94, 1.03)	0.97 (0.92, 1.01)	0.94 (0.90, 0.99)
	Heavy truck traffic (current)	1.17 (1.12, 1.22)	1.19 (1.14, 1.24)	1.12 (1.06, 1.17)
	Fast food (current)	0.99 (0.94, 1.03)	1.08 (1.04, 1.13)	0.99 (0.94, 1.05)
	Television (current)	0.93 (0.88, 0.98)	1.05 (0.99, 1.11)	0.96 (0.90, 1.02)
	Paternal tobacco (current)	1.07 (1.02, 1.12)	1.10 (1.05, 1.16)	1.04 (0.99, 1.10)
	Maternal tobacco (current)	1.05 (0.99, 1.11)	1.20 (1.14, 1.27)	1.05 (0.99, 1.12)
	Paracetamol (current)	2.02 (1.92, 2.13)	2.07 (1.97, 2.17)	1.46 (1.38, 1.55)
	Open fire cooking (current)	0.99 (0.82, 1.19)	1.21 (1.04, 1.42)	1.14 (0.96, 1.35)
				
13–14 years (n = 224,436)	2 or more siblings	1.04 (1.01, 1.07)	1.02 (0.99, 1.06)	1.08 (1.04, 1.12)
	Heavy truck traffic (current)	1.23 (1.20, 1.26)	1.20 (1.16, 1.23)	1.31 (1.26, 1.36)
	Fast food (current)	1.06 (1.03, 1.08)	1.07 (1.04, 1.10)	1.06 (1.02, 1.10)
	Television (current)	1.01 (0.97, 1.05)	1.02 (0.97, 1.07)	1.07 (1.01, 1.14)
	Paternal tobacco (current)	1.10 (1.07, 1.13)	1.11 (1.07, 1.14)	1.15 (1.11, 1.20)
	Maternal tobacco (current)	1.14 (1.10, 1.17)	1.22 (1.18, 1.27)	1.11 (1.06, 1.17)
	Paracetamol (current)	1.76 (1.71, 1.81)	1.80 (1.75, 1.86)	1.58 (1.52, 1.65)
	Open fire cooking (current)	1.16 (1.08, 1.26)	1.19 (1.10, 1.30)	1.49 (1.34, 1.65)

a Adjusted for sex, mothers level of education and for all other variables in the table (within age group).

b Synthesis sample contains individuals with data present for all 3 outcomes, sex, maternal education and all exposures of interest

For younger children, the strongest associations in the fully adjusted analyses were similar across all 3 outcomes. They were: current paracetamol use (ORs for rhinoconjunctivitis symptoms 2.02; asthma symptoms 2.07; and eczema symptoms 1.46), antibiotic use in the first year of life (1.58; 1.66; 1.40, respectively), and paracetamol use in the first year of life (1.39; 1.34; 1.29). Heavy truck traffic showed a less strong but consistent association with all 3 outcomes (1.17; 1.19; 1.12). Similarly, cat ownership in the first year of life had a consistent direction of association, somewhat stronger with asthma (1.22) than with rhinoconjunctivitis (1.09) and eczema (1.10).

The 3 diseases differed in their associations with some other risk factors. Exposures showing a harmful association with asthma but no statistically significant association with rhinoconjunctivitis and eczema were cooking on an open fire and fast food. Low birthweight showed a harmful association with asthma (OR = 1.15), a marginally statistically significant protective effect with eczema (OR = 0.90), and no association with rhinoconjunctivitis. Breast feeding was associated with increased risk of eczema (1.11), a marginally statistically significant protective association with asthma (OR = 0.95) and no association with rhinoconjunctivitis.

Among adolescents, exposures showing consistent associations with all 3 diseases were current paracetamol use (odds ratios for rhinoconjunctivitis 1.76; asthma 1.80; and eczema 1.58), heavy truck traffic (1.23; 1.20; 1.31, respectively), cooking on an open fire (1.16; 1.19; 1.49), mother smoking (1.14; 1.22; 1.11), and father smoking (1.10; 1.11; 1.15). Weaker associations with fast food were also consistent (1.06; 1.07; 1.06) across the 3 diseases (Table 3).

### Comparison of risk factor patterns in affluent and less affluent countries

**Fig. 1:**
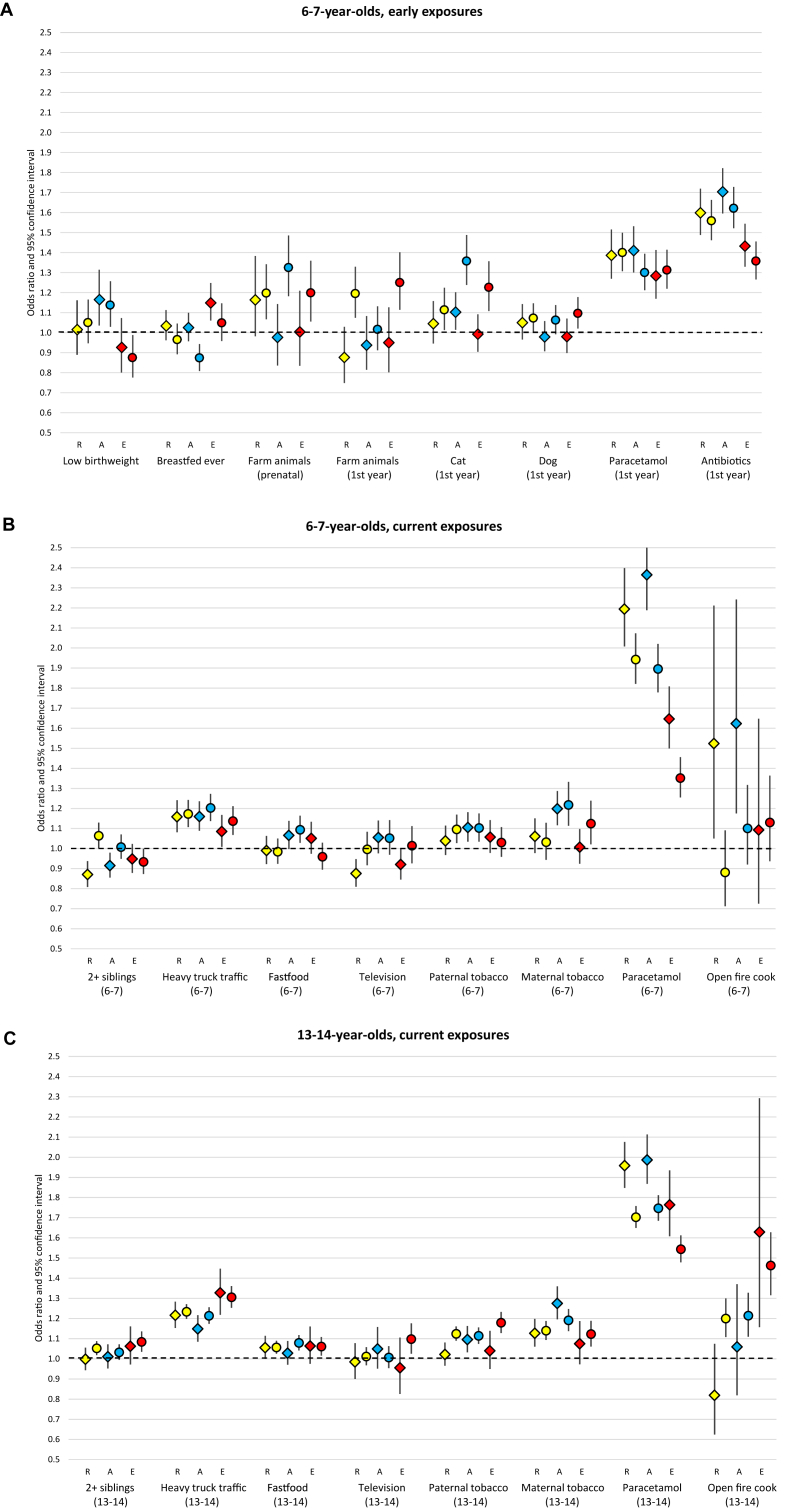
Mutually adjusted odds ratios and 95% confidence intervals for individual-level associations between risk factors and each of the three diseases, in affluent countries and non-affluent countries. 1A 6-7-year-olds, early exposures. 1B 6-7-year-olds, current exposures. 1C 13-14-year-olds, current exposures. Results from mixed logistic regression models with random intercepts at the school, centre and country levels. Adjusted for sex, mother's level of education and all other variables shown for the same age group. Based on the synthesis sample as shown in Table 3, stratified by country-level affluence. Results for affluent countries shown as diamonds (N = 41,831 aged 6–7; N = 46,932 aged 13–14). Results for non-affluent countries shown as circles (N = 75,032 aged 6–7; N = 177,504 aged 13–14). Results for rhino-conjunctivitis symptoms (R) shown in yellow. Results for asthma symptoms (A) shown in blue. Results for eczema symptoms (E) shown in red. Data points are tabulated in Supplementary Table S2.

Fig. 1A–C summarise the risk factor-disease associations by age group, stratified by national per capita GNI (Numerical results are shown in Table S2, Supporting Material.). Many of the risk factor-disease associations are fairly consistent between affluent and non-affluent settings, with most differences being within the range expected by chance (interaction p > 0.01). In the section below we focus upon the most significant inconsistencies (interaction p < 0.0001 for one or more diseases).

For rhinoconjunctivitis among 6-7-year-olds (Fig. 1A and B), notable differences by national per capita GNI are increased risk in non-affluent countries with farm animal contact in infancy (1.19; 1.07–1.33) and having more than two siblings (1.06; 1.00–1.13), whereas in affluent countries, these associations are protective (0.88; 0.75–1.03 and 0.87; 0.81–0.94, respectively).

For asthma among 6-7-year-olds (Fig. 1A and B), farm animal exposure in pregnancy showed a harmful association in non-affluent countries (1.32; 1.18–1.49) but no significant effect in affluent countries (0.98; 0.83–1.14). Breastfeeding ever showed a protective effect in non-affluent countries (0.87; 0.81–0.94) but no significant effect in affluent countries (1.02; 0.96–1.10). Cat exposure in infancy increased risk of asthma symptoms in both settings, but there was evidence of a stronger effect in non-affluent (1.36; 1.24–1.49) than affluent countries (1.10; 1.01–1.20). Conversely, current paracetamol use elevated asthma risk to a significantly greater extent in affluent (2.36; 2.19–2.56) than non-affluent settings (1.90; 1.78–2.02).

For eczema among 6-7-year-olds (Fig. 1A and B), early cat exposure was a risk factor in non-affluent countries (1.23; 1.11–1.36) but not in affluent countries (0.99; 0.90–1.09). Farm animal exposure in infancy increased eczema risk in non-affluent (1.25; 1.11–1.40) but not in affluent countries (0.95; 0.80–1.13), and a similar pattern was evident for farm animal exposure in pregnancy. Current paracetamol use was more strongly associated with eczema symptoms in affluent (1.65; 1.50–1.81) than non-affluent settings (1.35; 1.25–1.46), although this heterogeneity (interaction p = 0.003) was less significant than for asthma.

Stratifying the 13-14 year-old results in a similar manner (Fig. 1C and Table S2), few risk factors demonstrate differential effects in affluent and non-affluent countries. For rhinoconjunctivitis, open fire cooking increased risk in non-affluent countries (1.20; 1.11–1.30) but not in affluent countries (0.82; 0.62–1.07) (interaction p = 0.008). Across all 3 outcomes, current paracetamol use showed a harmful effect in both affluent and non-affluent countries, but the effect was stronger in affluent countries (interaction p < 0.0001 for rhinoconjunctivitis, p = 0.0009 for asthma, p = 0.009 for eczema).

### Multimorbid (combinations of disease) models

In the 6-7 year-old synthesis sample, 80.1% of the children had no symptoms of any of the 3 outcomes. The proportion of children with only 1 disease was 14.9% (rhinoconjunctivitis 4.7%, asthma 5.8%, eczema 4.4%). The proportions with 2 diseases was 4.1% (rhinoconjunctivitis and asthma 2.1%, rhinoconjunctivitis and eczema 1.1%, and asthma, and eczema 0.9%). Just 0.9% of the sample had symptoms of all 3 diseases (Table 1).

Using models comparing different combinations of disease outcomes to those with no disease (Table 4), we found that antibiotics in the first year of life showed a stronger effect among individuals with 2 or 3 diseases. Paracetamol in the first year had similar effects across any combination of the diseases, with a slightly stronger effect only noticed with individuals who have all 3 diseases. This was similar for current heavy truck traffic. Current paracetamol showed a stronger effect in asthma and rhinoconjunctivitis than eczema, as reflected in the combinations of multiple diseases with the strongest effects being in individuals with all 3 diseases or rhinoconjunctivitis and asthma (Table 4).

**Table 4:** Multi outcome models of fully adjusted^a^ within school effects of exposures compared to a reference group with no disease. Mixed logistic regression models with random intercepts at the school, centre and country levels.

	RC only	Asthma only	Eczema only	RC and Asthma	RC and Eczema	Asthma and Eczema	RC, Asthma and Eczema
Exposures of interest	OR (95% CI)	OR (95% CI)	OR (95% CI)	OR (95% CI)	OR (95% CI)	OR (95% CI)	OR (95% CI)
Age 6–7 years	Reference group with no disease, n_0_ = 93,554						
	n_1_ = 5508	n_1_ = 6720	n_1_ = 5099	n_1_ = 2503	n_1_ = 1310	n_1_ = 1074	n_1_ = 1095
Low birthweight	0.98 (0.88, 1.10)	1.16 (1.05, 1.27)	0.85 (0.75, 0.96)	1.14 (0.98, 1.33)	0.96 (0.76, 1.20)	0.90 (0.70, 1.16)	1.14 (0.91, 1.44)
Breastfed ever	0.98 (0.91, 1.05)	0.93 (0.87, 1.00)	1.12 (1.03, 1.21)	0.96 (0.86, 1.06)	1.10 (0.95, 1.28)	0.98 (0.84, 1.15)	1.12 (0.96, 1.32)
Farm animals (prenatal)	1.17 (1.03, 1.34)	1.14 (1.01, 1.28)	1.06 (0.93, 1.22)	1.22 (1.01, 1.47)	1.07 (0.84, 1.37)	1.19 (0.89, 1.58)	1.56 (1.20, 2.03)
Farm animals (1st year)	1.11 (0.99, 1.25)	1.03 (0.92, 1.15)	1.17 (1.03, 1.32)	0.97 (0.82, 1.16)	1.40 (1.13, 1.74)	1.04 (0.80, 1.35)	0.86 (0.66, 1.12)
Cat (1st year)	0.97 (0.88, 1.07)	1.21 (1.12, 1.31)	1.08 (0.99, 1.18)	1.29 (1.14, 1.47)	1.19 (1.00, 1.42)	1.06 (0.89, 1.27)	1.16 (0.97, 1.39)
Dog (1st year)	1.07 (1.00, 1.15)	1.01 (0.94, 1.08)	1.07 (1.00, 1.15)	1.08 (0.98, 1.20)	1.05 (0.92, 1.20)	0.99 (0.86, 1.15)	1.14 (0.99, 1.32)
Paracetamol (1st year)	1.38 (1.29, 1.48)	1.32 (1.24, 1.41)	1.26 (1.17, 1.35)	1.45 (1.30, 1.62)	1.49 (1.29, 1.72)	1.35 (1.14, 1.60)	1.82 (1.52, 2.18)
Antibiotics (1st year)	1.44 (1.36, 1.54)	1.56 (1.47, 1.65)	1.25 (1.17, 1.33)	1.95 (1.77, 2.15)	1.82 (1.60, 2.07)	2.13 (1.84, 2.47)	2.37 (2.03, 2.76)
2 or more siblings	0.98 (0.92, 1.04)	0.96 (0.90, 1.01)	0.94 (0.88, 1.00)	0.98 (0.90, 1.08)	0.91 (0.81, 1.03)	0.88 (0.77, 1.01)	0.99 (0.86, 1.13)
Heavy truck traffic (current)	1.13 (1.06, 1.20)	1.18 (1.12, 1.24)	1.08 (1.01, 1.15)	1.19 (1.09, 1.29)	1.15 (1.02, 1.29)	1.11 (0.98, 1.26)	1.50 (1.32, 1.71)
Fast food (current)	0.96 (0.90, 1.02)	1.08 (1.02, 1.15)	0.96 (0.90, 1.03)	1.06 (0.97, 1.16)	1.01 (0.89, 1.14)	1.21 (1.05, 1.38)	0.98 (0.86, 1.13)
Television (current)	0.91 (0.85, 0.98)	1.07 (0.99, 1.15)	0.97 (0.90, 1.05)	1.02 (0.91, 1.14)	0.85 (0.73, 0.99)	1.05 (0.88, 1.25)	0.95 (0.80, 1.12)
Paternal tobacco (current)	1.05 (0.98, 1.12)	1.08 (1.02, 1.15)	1.02 (0.96, 1.10)	1.12 (1.03, 1.23)	0.93 (0.82, 1.06)	1.12 (0.97, 1.29)	1.22 (1.06, 1.40)
Maternal tobacco (current)	1.02 (0.94, 1.11)	1.24 (1.16, 1.33)	1.05 (0.97, 1.14)	1.21 (1.09, 1.36)	1.11 (0.94, 1.30)	1.30 (1.11, 1.53)	1.05 (0.89, 1.24)
Paracetamol (current)	1.90 (1.77, 2.04)	1.96 (1.84, 2.08)	1.34 (1.24, 1.45)	2.86 (2.59, 3.15)	1.91 (1.66, 2.19)	2.17 (1.88, 2.51)	2.92 (2.53, 3.37)
Open fire cooking (current)	0.97 (0.75, 1.25)	1.28 (1.07, 1.55)	1.18 (0.96, 1.45)	1.17 (0.83, 1.67)	1.09 (0.70, 1.72)	1.21 (0.77, 1.91)	0.77 (0.43, 1.39)

	RC only	Asthma only	Eczema only	RC and Asthma	RC and Eczema	Asthma and Eczema	RC, Asthma and Eczema
Exposures of interest	OR (95% CI)	OR (95% CI)	OR (95% CI)	OR (95% CI)	OR (95% CI)	OR (95% CI)	OR (95% CI)
Age 13–14 years	Reference group with no disease, n_0_ = 170,542						
	n_1_ = 19,858	n_1_ = 13,585	n_1_ = 7104	n_1_ = 6557	n_1_ = 3219	n_1_ = 1554	n_1_ = 2017
2 or more siblings	1.02 (0.98, 1.06)	1.01 (0.97, 1.05)	1.06 (1.00, 1.12)	1.05 (0.99, 1.11)	1.18 (1.09, 1.29)	1.06 (0.95, 1.19)	1.12 (1.01, 1.24)
Heavy truck traffic (current)	1.19 (1.16, 1.23)	1.14 (1.09, 1.18)	1.26 (1.20, 1.33)	1.30 (1.23, 1.37)	1.44 (1.34, 1.55)	1.47 (1.33, 1.64)	1.56 (1.42, 1.71)
Fast food (current)	1.04 (1.01, 1.08)	1.06 (1.02, 1.10)	1.06 (1.00, 1.12)	1.13 (1.07, 1.19)	1.12 (1.04, 1.21)	1.13 (1.01, 1.26)	1.13 (1.02, 1.24)
Television (current)	1.03 (0.98, 1.08)	1.08 (1.02, 1.15)	1.09 (1.01, 1.19)	0.97 (0.89, 1.06)	1.23 (1.08, 1.41)	1.21 (1.01, 1.46)	0.80 (0.69, 0.92)
Paternal tobacco (current)	1.08 (1.04, 1.11)	1.10 (1.05, 1.14)	1.13 (1.07, 1.19)	1.14 (1.07, 1.20)	1.22 (1.13, 1.32)	1.16 (1.04, 1.30)	1.26 (1.14, 1.39)
Maternal tobacco (current)	1.09 (1.05, 1.14)	1.20 (1.15, 1.26)	1.07 (0.99, 1.14)	1.27 (1.19, 1.35)	1.19 (1.08, 1.31)	1.22 (1.06, 1.39)	1.32 (1.18, 1.49)
Paracetamol (current)	1.62 (1.56, 1.68)	1.69 (1.62, 1.76)	1.36 (1.29, 1.44)	2.34 (2.21, 2.47)	2.16 (2.00, 2.34)	2.12 (1.89, 2.36)	2.98 (2.71, 3.29)
Open fire cooking (current)	1.15 (1.05, 1.27)	1.12 (1.01, 1.25)	1.33 (1.15, 1.53)	1.09 (0.92, 1.28)	1.50 (1.22, 1.84)	1.93 (1.49, 2.50)	2.22 (1.76, 2.81)

a Adjusted for sex, mother's level of education and all other variables in the table for that age group

Among the 13-14 year-old synthesis sample, 76.0% had no symptoms of any of the 3 outcomes. The proportion of adolescents with only 1 disease was 18.1% (rhinoconjunctivitis 8.8%, asthma 6.1%, eczema 3.2%). A further 5.0% had symptoms of 2 of the diseases (rhinoconjunctivitis and asthma 2.9%, rhinoconjunctivitis and eczema 1.4% and asthma and eczema 0.7%). Only 0.9% had symptoms of all 3 diseases (Table 1).

Current paracetamol showed a stronger effect in individuals with more than one disease, with the strongest effect in those with all 3 diseases. Open fire cooking showed a stronger effect in all combinations that contain eczema (Table 4).

Importantly, in both age groups, risk factor associations with each disease in the whole population (Table 3) persisted among children with only 1 condition, after exclusion of multimorbid groups (Table 4).

### Unimorbid (single disease case-only) models

Table 5 shows the results of 3 separate models, each comparing 2 of the unimorbid outcomes. Corresponding results, stratified by per capita GNI, are shown as Table S3 in the Supporting Material. Triangle plots appear as Figs. S3–S8 in the Supporting Material. An equilateral central triangle denotes a risk factor that has a similar strength of effect on all 3 diseases, the further from equilateral the triangle is, the more that risk factor effect differs in strength between diseases. In the plots the odds ratios displayed are all greater than or equal to one; they relate to whichever disease has the stronger effect (the corner they are closest to compared to the opposite corner).

**Table 5:** Fully adjusted^a^ unimorbid two-way models. Mixed logistic regression models with random intercepts at the school, centre and country levels.

Age group	Exposures of Interest	Asthma v Eczema	Asthma v Rhinoconjunctivitis	Rhinoconjunctivitis v Eczema
		OR (95% CI)	OR (95% CI)	OR (95% CI)
6–7 years		n = 11,819	n = 12,228	n = 10,607
	Low birthweight	1.40 (1.19, 1.64)	1.16 (0.99, 1.35)	1.11 (0.94, 1.32)
	Breastfed ever	0.85 (0.76, 0.94)	0.94 (0.85, 1.05)	0.89 (0.80, 1.00)
	Farm animals (prenatal)	1.03 (0.85, 1.23)	0.99 (0.82, 1.18)	1.04 (0.86, 1.26)
	Farm animals (1st year)	0.90 (0.76, 1.06)	0.89 (0.75, 1.05)	0.98 (0.83, 1.17)
	Cat (1st year)	1.12 (0.99, 1.26)	1.27 (1.11, 1.44)	0.83 (0.73, 0.96)
	Dog (1st year)	0.94 (0.85, 1.04)	0.97 (0.87, 1.07)	0.95 (0.86, 1.05)
	Paracetamol (1st year)	1.05 (0.95, 1.16)	0.96 (0.86, 1.06)	1.07 (0.96, 1.19)
	Antibiotics (1st year)	1.26 (1.16, 1.38)	1.12 (1.03, 1.23)	1.14 (1.03, 1.25)
	2 or more siblings	1.01 (0.93, 1.10)	0.99 (0.90, 1.08)	1.03 (0.94, 1.13)
	Heavy truck traffic (current)	1.08 (1.00, 1.17)	1.05 (0.97, 1.14)	1.06 (0.97, 1.16)
	Fast food (current)	1.12 (1.02, 1.22)	1.12 (1.03, 1.23)	1.00 (0.91, 1.10)
	Television (current)	1.13 (1.01, 1.26)	1.16 (1.04, 1.29)	0.92 (0.82, 1.03)
	Paternal tobacco (current)	1.03 (0.94, 1.12)	1.03 (0.94, 1.13)	1.02 (0.92, 1.12)
	Maternal tobacco (current)	1.16 (1.04, 1.29)	1.21 (1.08, 1.35)	0.94 (0.84, 1.07)
	Paracetamol (current)	1.45 (1.31, 1.60)	1.03 (0.93, 1.14)	1.48 (1.32, 1.65)
	Open fire cooking (current)	1.02 (0.77, 1.35)	1.32 (0.95, 1.83)	0.72 (0.51, 1.01)
13–14 years		n = 20,689	n = 33,443	n = 26,962
	2 or more siblings	0.94 (0.87, 1.01)	0.99 (0.94, 1.05)	0.96 (0.90, 1.03)
	Heavy truck traffic (current)	0.92 (0.86, 0.99)	0.97 (0.92, 1.02)	0.93 (0.87, 0.98)
	Fast food (current)	1.01 (0.94, 1.08)	0.99 (0.94, 1.04)	1.00 (0.94, 1.06)
	Television (current)	0.98 (0.88, 1.09)	1.06 (0.98, 1.15)	0.96 (0.87, 1.06)
	Paternal tobacco (current)	0.96 (0.89, 1.03)	1.01 (0.96, 1.06)	0.96 (0.90, 1.03)
	Maternal tobacco (current)	1.15 (1.06, 1.26)	1.13 (1.06, 1.21)	1.02 (0.94, 1.11)
	Paracetamol (current)	1.17 (1.09, 1.26)	1.02 (0.97, 1.08)	1.16 (1.09, 1.24)
	Open fire cooking (current)	0.89 (0.74, 1.06)	1.05 (0.91, 1.21)	0.80 (0.68, 0.95)

a Adjusted for sex, mother's level of education and all other variables in the table for that age group

In the 6-7 year-old age group, low birthweight was most strongly associated with asthma and more strongly associated with rhinoconjunctivitis than eczema. Early life antibiotic exposure showed a similar pattern but to a slightly reduced extent. Being breastfed ever showed a stronger association with eczema than both asthma and rhinoconjunctivitis. Owning a cat in the first year of life was most strongly associated with asthma but more strongly associated with eczema than rhinoconjunctivitis.

Some differences were evident between affluent and non-affluent countries (Table S3 and Fig. S6). Farm animal contact during pregnancy had effects in non-affluent countries which were more balanced between the diseases, but in affluent countries the effect was stronger on eczema and rhinoconjunctivitis than asthma. In contrast, early cat contact had more balanced effects in affluent countries, but in non-affluent countries there was a much stronger effect on asthma and eczema than rhinoconjunctivitis.

Among the current exposures for 6-7 year-olds, current paracetamol use was more strongly associated with asthma and rhinoconjunctivitis than with eczema. Open fire cooking was more strongly associated with both asthma and eczema than rhinoconjunctivitis but the confidence intervals were wide. Maternal smoking was more strongly associated with asthma than with eczema or rhinoconjunctivitis. Affluent centres showed a stronger effect of open fire cooking on asthma and rhinoconjunctivitis, whereas in non-affluent centres the stronger effect was on asthma and eczema.

Among 13-14 year-olds, similar to the younger children, maternal smoking had a stronger association with asthma than with either eczema or rhinoconjunctivitis, and current paracetamol showed a stronger association with asthma and rhinoconjunctivitis than with eczema. The biggest difference between affluent and non-affluent countries was observed for open fire cooking. In affluent countries, there were stronger associations with eczema than with asthma or rhinoconjunctivitis, although it is important to note that the confidence intervals were exceptionally large due to the rarity of cooking on open fires in the affluent centres.

## Discussion

### Overview of findings

This is the largest and broadest overview to date of lifestyle and environmental risk factors for symptoms of non-infective rhinoconjunctivitis among children. It is the first comprehensive analysis of this condition, which models multiple risk factors together to compare their mutually adjusted individual-level and population (school)-level associations in a multilevel framework. Due to the multiple comparisons made, and the large size of our sample, we concentrate our interpretation upon the overall patterns of results and on specific findings with more extreme levels of statistical significance.

Generally, associations with exposures averaged at the school level were similar in direction and magnitude to those ascertained at the child level, as we found also for symptoms of asthma and eczema. As we have argued elsewhere,^20^^,^^21^ this helps to exclude reverse causation, particularly for exposures such as early paracetamol and antibiotic use which may be related to prodromal disease, or pets which may be avoided by allergic families. An exception are the results for breastfeeding, showing a borderline significant inverse association with asthma symptoms, a significantly positive association with eczema symptoms, and a null association with rhinoconjunctivitis at the individual level (Table 3). Nevertheless, the association of breastfeeding with rhinoconjunctivitis at the school level is strongly and significantly inverse (Table 2), perhaps indicating confounding by socioeconomic or other unmeasured characteristics of the school catchment population. This contrasts with the pattern of school-level associations of breastfeeding with symptoms of asthma^20^ and eczema,^21^ which were weakly positive but non-significant.

Many of the risk factor associations observed for symptoms of rhinoconjunctivitis were similar to those previously reported for symptoms of asthma or eczema in ISAAC Phase Three.^6^, ^7^, ^8^, ^9^, ^10^, ^11^, ^12^, ^13^, ^14^, ^15^, ^16^, ^17^, ^18^, ^19^, ^20^, ^21^ Since the 3 diseases cluster together at the individual level, it is possible that associations observed for one disease could be influenced by risk factors for other conditions in the triad. An innovative use of ISAAC data in this paper is the analysis of rhinoconjunctivitis, asthma and eczema, singly and in combination.

As expected, we found that associations with multimorbidity (combinations of 2 or 3 diseases) were stronger than for each disease alone (unimorbidity). However, the relationships of risk factors with each disease in the absence of the others were of similar direction and magnitude to the results for each condition modelled separately. Thus, multimorbidity is not the sole explanation of the common epidemiological patterns across these three diseases.

For many risk factors, associations were consistent across the 3 diseases, between the 2 age groups, and between countries with different levels of per capita Gross National Income. This similarity of epidemiology strongly suggests that there are common biological mechanisms for these 3 diseases, which operate in both affluent and less affluent settings. The most striking example of this in our ISAAC dataset is current paracetamol exposure, which was consistently associated with each of the 3 diseases, within schools and between schools, in both age groups and in richer and poorer countries, although somewhat more strongly with rhinoconjunctivitis and asthma than with eczema.

Shared mechanisms do not exclude the possibility of disease-specific pathways, which may differ between higher and lower income countries. An example of the latter is the inverse association of seasonal rhinoconjunctivitis with number of siblings and childhood exposure to the farm environment. This is well established from large epidemiological studies in Europe and confirmed by objective markers of allergic sensitisation.^26^ This pattern is consistent with our findings for rhinoconjunctivitis symptoms in affluent countries but contrasts with the increased risk of these symptoms among children from larger families and those exposed to farm animals in poorer countries.

### Strengths and limitations

ISAAC Phase Three has substantial advantages in terms of large sample sizes drawn from diverse study centres worldwide, who adopted standardised methods of data collection. Reliance solely upon questionnaires completed by parents (for the 6-7-year-olds) or participants (for the 13-14-year-olds) is a limitation, both for definition of disease outcomes and for ascertainment of risk factors. On the other hand, the questionnaire methodology maintained high response rates in each centre.

Misclassification of disease or risk factor information could be non-systematic, leading to weaker associations, or systematic, potentially exaggerating, or masking associations. A particular concern would be individual differences in the threshold for reporting of symptoms, which could exaggerate clustering of the 3 complaints within individual children. This is unlikely to affect risk factor associations in our unimorbid analysis, where similarity in the epidemiological patterns for the each of the 3 diseases (in the absence of the others, Table 4) would be biased only if reporting of all 3 diseases were altered by the presence of the risk factor (or conversely, reporting of the common risk factor were biased to a similar degree by the presence of each of the 3 diseases).

A particular limitation of ISAAC Phase Three is the lack of objective information on allergic sensitisation. This was measured by skin prick testing and serum allergen-specific IgE in a separate study of more than 50,000 10-11-year-old children in 30 centres from 22 countries (ISAAC Phase Two).^3^, ^4^, ^5^^,^^27^ Although (as expected) symptoms of rhinoconjunctivitis, asthma and eczema were more common among children with positive skin prick tests, these associations were substantially weaker in less affluent settings. The proportion of each disease attributable to skin prick positivity in centres from lower-income countries (per capita GNI < USD 9200 in 2001) was 14% for rhinoconjunctivitis, 20% for asthma but only 1% for eczema. The corresponding population attributable fractions (PAFs) in higher-income countries were 61% for rhinoconjunctivitis, 46% for asthma, and 28% for eczema. The PAF estimates were very similar when serum allergen-specific IgE was used as the measure of allergic sensitisation.^4^

Throughout, ISAAC has focused upon the combination of nasal and conjunctival symptoms (in the absence of intercurrent infection) as the most relevant definition of rhinoconjunctivitis because non-infective rhinitis alone (without itchy eyes) is less strongly associated with skin prick positivity (PAFs of 5% in less affluent centres and 10% in more affluent centres).^27^ More recent studies, among adolescents^28^ and adults^29^ have confirmed a stronger association of allergic sensitisation with rhinoconjunctivitis than with rhinitis alone.

### A shared non-allergic mechanism for “atopic diseases”?

In recent decades, two models of multimorbidity have been proposed to explain the occurrence of 2 or more “atopic diseases” (asthma, rhinitis, and eczema, sometimes extended also to food allergy). The term “united airway disease” has been proposed for the coexistence of asthma and rhinitis in the same patient at the same time.^30^, ^31^, ^32^ The concept of the “atopic march” applies to the sequential development of eczema, asthma, and rhinitis (usually in that order) through childhood and adolescence.^33^^,^^34^ Discussion of both concepts has tended to focus upon IgE-mediated allergic mechanisms and Th2-immune inflammatory pathways as an explanation for concurrent and longitudinal clustering of the 3 diseases.

However, it is recognised that several distinct pathways and mechanisms are likely to be involved in the atopic march, some of them common and some disease-specific.^34^ Non-allergic airway inflammation, defects of mucosal defence, and exogenous cofactors (including microbes, pollutants and smoking) have been proposed as “treatable traits” underlying united airway disease,^32^ in addition to the close association of both asthma and rhinitis with IgE sensitisation, particularly to multiple allergens.^35^

Genome-wide association studies have shown a mixture of common and disease-specific signals for asthma, hay fever, and eczema^36^ illustrated by triangle plots derived from unimorbid case-only comparisons similar to those we have shown in Table 5 and Figs. S3–S8. Filaggrin (*FLG*) variants are specifically associated with eczema, whereas other genome-wide significant loci such as *IL6R* show almost perfect symmetry in association with each of the 3 diseases. A bioinformatics (data-mining) analysis of protein interactions found that asthma, rhinitis, and eczema shared more associated proteins than would be expected by chance and identified 15 pathways potentially involved in the multimorbidity of asthma, rhinitis, and eczema, although many of these are related to Th2-immune signalling pathways.^37^

Epidemiological evidence for non-allergic mechanisms underlying coexistence of asthma, rhinitis, and eczema emerges from the collaborative analysis of European birth cohorts by the MeDALL consortium.^38^ Among over 8000 children followed from birth to 4 and 8 years of age, IgE sensitisation to common food and aero-allergens at age 4 years accounted for only 38% of the co-occurrence of 2 or more conditions (asthma, rhinitis, and eczema) at age 8 years. In relative terms, the strength of the association among the 3 diseases was higher in non-sensitised children, although the excess comorbidity was greater among those who were sensitised, due to a higher baseline risk of disease. Among the sensitised children, about a quarter of the observed comorbidity was not due to chance, whereas among non-sensitised children the non-chance proportion was more than half at age 8 years. Comorbidity at age 4 years was strongly predictive of comorbidity at age 8 years. All these observations led the MeDALL investigators to propose “a new vision of multimorbidity independent of IgE sensitisation”,^39^ which would be entirely consistent with our observations of common epidemiological patterns for symptoms of rhinoconjunctivitis, asthma, and eczema in 2 age groups of children in both affluent and non-affluent countries worldwide.

## Conclusions

Most of the environmental and lifestyle correlates of rhinoconjunctivitis, asthma, and eczema in childhood display similarity across the 3 conditions, even in less affluent settings where allergic sensitisation (as conventionally defined) is less likely to explain the concordant epidemiological patterns. This supports the view that mechanisms other than IgE-mediated tissue inflammation may contribute a substantial proportion of the clustering of these “atopic diseases” within individuals (concurrently and sequentially) and at the population level.

## Author contributions

Data collection was co-ordinated by I.A., P.E., N.P., L.G-M. and D.S. and implemented by the ISAAC Phase Three Study Group. This data analysis and presentation were conceived by C.R., R.S., N.P. and D.S. Statistical modelling was performed by C.R. as part of PhD studies supervised by R.S., N.P. and D.S. The manuscript was drafted by C.R. and D.S. and critically reviewed by all named authors.

## Financial disclosure

We would like to acknowledge and thank the many funding bodies throughout the world that supported the individual ISAAC centres and collaborators and their meetings. In particular, we wish to thank the London School of Hygiene and Tropical Medicine, and the United Kingdom Medical Research Council for supporting the work involved in the current paper. We also wish to thank the Health Research Council of New Zealand, the Asthma and Respiratory Foundation of New Zealand, the Child Health Research Foundation, the Hawke's Bay Medical Research Foundation, the Waikato Medical Research Foundation, Glaxo Wellcome New Zealand, the NZ Lottery Board, and Astra Zeneca New Zealand. Glaxo Wellcome International Medical Affairs supported the Regional Coordination and the ISAAC International Data Centre (IIDC). Without help from all of the above, ISAAC would not have given us all these results from so many countries.

## Funding

This work was supported by the 10.13039/501100000265United Kingdom Medical Research Council [grant number MR/N013638/1] and the 10.13039/100010663European Research Council under the European Union's Seventh Framework Programme [FP7/2007–2013/ERC grant agreement number 668954]. The Centre for Global NCDs is supported by the 10.13039/100004440Wellcome Trust Institutional Strategic Support Fund [097834/Z/11/B].

## Role of the funding sources

The funders had no involvement in the conduct of the research (collection, analysis or preparation of data), nor in the writing of this report, nor in the decision to submit the article for publication.

## Consent for publication

No additional consent (from institutions or funders) is required for publication.

## Ethical approval

Not applicable, as this is secondary analysis of an existing, publicly available dataset.

## Availability of data

The ISAAC Phase Three datasets, on which this article is based, and associated survey documentation, have been deposited at the UK Data Archive for use by *bona fide* researchers on application via the following URL: http://discover.ukdataservice.ac.uk/catalogue?sn=8131 (https://doi.org/10.5255/UKDA-SN-8131-1)

## Declaration of competing interest

The authors report no competing interests.

## Appendix

### ISAAC Phase Three Study Group

***ISAAC Steering Committee:*** N Aït-Khaled∗ (Union Internationale Contre la Tuberculose et les Maladies Respiratoires, Paris, France); HR Anderson (Population Health Research Institute, St George's, University of London, UK); MI Asher (Department of Paediatrics: Child and Youth Health, Faculty of Medical and Health Sciences, The University of Auckland, New Zealand); R Beasley∗ (Medical Research Institute of New Zealand, Wellington, New Zealand); B Björkstén∗ (Institute of Environmental Medicine, Karolinska Institutet, Stockholm, Sweden); B Brunekreef (Institute of Risk Assessment Science, Universiteit Utrecht, Netherlands); J Crane (Wellington Asthma Research Group, Wellington School of Medicine, New Zealand); P Ellwood (Department of Paediatrics: Child and Youth Health, Faculty of Medical and Health Sciences, The University of Auckland, New Zealand); C Flohr (Unit for Population-Based Dermatology Research, St John's Institute of Dermatology, Guy's and St Thomas' NHS Foundation Trust and King's College London, London, UK); S Foliaki∗ (Centre for Public Health Research, Massey University, Wellington, New Zealand); F Forastiere (Department of Epidemiology, Rome E Health Authority, Italy); L García-Marcos (Pediatric Allergy and Pulmonology Units, Virgen de la Arrixaca University Children's Hospital, University of Murcia and Bio-health Research Institute of Murcia (IMIB), Murcia, Spain); U Keil∗ (Institut für Epidemiologie und Sozialmedizin, Universität Münster, Germany); CKW Lai∗ (Department of Medicine and Therapeutics, The Chinese University of Hong Kong, SAR China); J Mallol∗ (Department of Respiratory Medicine, University of Santiago de Chile, Chile); EA Mitchell (Department of Paediatrics, Faculty of Medical and Health Sciences, The University of Auckland, New Zealand); S Montefort∗ (Department of Medicine, University of Malta, Malta), J Odhiambo†∗ (Centre Respiratory Diseases Research Unit, Kenya Medical Research Institute, Nairobi, Kenya); N Pearce (Centre for Public Health Research, Massey University, Wellington, New Zealand); CF Robertson (Murdoch Children's Research Institute, Melbourne, Australia); AW Stewart (Population Health, Faculty of Medical and Health Sciences, The University of Auckland, New Zealand); D Strachan (Population Health Research Institute, St George's, University of London, UK); E von Mutius (Dr von Haunerschen Kinderklinik de Universität München, Germany); SK Weiland† (Department of Epidemiology, University of Ulm, Germany); G Weinmayr (Institute of Epidemiology, University of Ulm, Germany); H Williams (Centre for Evidence Based Dermatology, Queen's Medical Centre, University Hospital, Nottingham, UK); G Wong (Department of Paediatrics, Prince of Wales Hospital, Hong Kong, SAR China).

∗Regional Coordinators. †Deceased.

***ISAAC International Data Centre:*** MI Asher, TO Clayton†, E Ellwood, P Ellwood, EA Mitchell, Department of Paediatrics: Child and Youth Health, and AW Stewart, School of Population Health, Faculty of Medical and Health Sciences, The University of Auckland, New Zealand.

†Deceased.

***ISAAC Principal Investigators:* Argentina:** CE Baena-Cagnani∗†, Catholic University of Córdoba (Córdoba), M Gómez, Ayre Foundation; Hospital San Bernardo (Salta); **Barbados:** ME Howitt∗, Carlton Clinic (Barbados); **Belgium:** J Weyler, University of Antwerp (Antwerp); **Bolivia:** R Pinto-Vargas∗, Caja Petrolera de Salud (Santa Cruz); **Brazil:** AJLA da Cunha, Federal University of Rio de Janeiro (Nova Iguaçu), L de Freitas Souza, Universidade Federal da Bahia (Feira de Santana, Salvador, Vitória da Conquista); **Cameroon:** C Kuaban∗, University of Yaounde (Yaounde); **Canada:** A Ferguson, University of British Columbia (Vancouver), D Rennie, University of Saskatchewan (Saskatoon); **Channel Islands:** P Standring, Princess Elizabeth Hospital (Guernsey); **Chile:** P Aguilar, Hospital CRS El Pino (South Santiago), L Amarales, Regional Hospital “Lautaro Navarro” (Punta Arenas), LA Benavides, (Calama), A Contreras, Hospital de Castro (Chiloe); **China:** Y-Z Chen∗, Training Hospital for Peking University (Beijing, Tong Zhou), O Kunii, University of Tokyo (Tibet), Q Li Pan, Xinjiang Children's Hospital (Wulumuqi), NS Zhong, Guangzhou Institute of Respiratory Disease (Guangzhou); **Colombia:** G Aristizábal, Instituto de Enfermedades Respiratorias del Niño S.A. (Bogotá), AM Cepeda, Universidad Metropolitana (Barranquilla), GA Ordoñez, Universidad Libre de Cali (Cali); **Ecuador:** C Bustos, Hospital Alcivar (Guayaquil); **Estonia:** M-A Riikjärv∗, Tallinn Children's Hospital (Tallinn); **Ethiopia:** K Melaku, Addis Ababa University (Addis Ababa); **Fiji:** R Sa'aga-Banuve, UNICEF (Suva); **Finland:** J Pekkanen∗, National Public Health Institute (Kuopio County); **Gabon:** IE Hypolite∗, (Port-Gentil); **Hungary:** Z Novák, University of Szeged (Szeged), G Zsigmond∗, Senior Consultant (Svábhegy); **India:** S Awasthi, King George's Medical University (Lucknow), S Bhave, KEM Hospital Research Centre (Rasta Peth), NM Hanumante, Ruby Hall Clinic (Pune), KC Jain, Pioneer Medical Centre (Jodhpur), MK Joshi, Panjat Hospital (Mumbai (16)), VA Khatav, Dr Khatav's Mother and Child Hospital (Borivali), SN Mantri, Jaslok Hospital & Research Centre (Mumbai (29)), AV Pherwani, P.D. Hinduja Hospital and Medical Research Centre (Mumbai (18)), S Rego, St John's Medical College & Hospital (Bangalore), M Sabir, Maharaja Agrasen Medical College Agroha (Bikaner), S Salvi, Chest Research Foundation (Nagpur, Pimpri), G Setty, (Chennai), SK Sharma, All India Institute of Medical Sciences (New Delhi (7)), V Singh, Asthma Bhawan (Jaipur), T Sukumaran, Pushpagiri Institute of Medical Sciences and Research Center [PIMS], Thiruvalla (Kottayam); **Indonesia:** CB Kartasasmita, Padjajaran University (Bandung), P Konthen†, Airlangga University (Bali), W Suprihati, Diponegoro University (Semarang); **Iran:** MR Masjedi∗, National Research Institute of Tuberculosis and Lung Diseases (Rasht,Tehran); **Isle of Man:** A Steriu, Freelance Public Health Consultant, Carol Davila Medical School, Bucharest (Isle of Man); **Ivory Coast:** BN Koffi∗, (Urban Cote d Ivoire); **Kuwait:** JA al-Momen, Al-Amiri Hospital (Kuwait); **Kyrgyzstan:** C Imanalieva∗, Kyrgyz Scientific Research Institute of Obstetrics and Pediatrics (Balykchi, Bishkek); **Lithuania:** J Kudzyte∗, Kaunas Medical University (Kaunas); **Malaysia:** BS Quah, Melaka-Manipal Medical College, (Kota Bharu), KH Teh, Hospital Alor Setar (Alor Setar); **Malta:** S Montefort∗, University of Malta (Malta); **Mexico:** M Baeza-Bacab∗, University Autónoma de Yucatán (Mérida), M Barragán-Meijueiro, Colegio Mexicano de Alergia, Asma e Inmunología Pediátrica [CoMAAIPE] (Ciudad de México (3)), BE Del-Río-Navarro, Hospital Infantil de México (Ciudad de México (1)), R García-Almaráz, Hospital Infantil de Tamaulipas (Ciudad Victoria), SN González-Díaz, Hospital Universitario (Monterrey), FJ Linares-Zapién, Centro De Enfermedades Alergicas Y Asma de Toluca (Toluca), JV Merida-Palacio, Centro de Investigacion de Enfermedades Alergicas y Respiratorias (Mexicali Valley), N Ramírez-Chanona, Colegio Mexicano de Pediatras Especialistas en Inmunología Clínica y Alergia [COMPEDIA] (Ciudad de México (4)), S Romero-Tapia, Hospital de Alta Especialidad del Niño (Villahermosa), I Romieu, International Agency for Research on Cancer (Cuernavaca); **Morocco:** Z Bouayad∗, Service des Maladies Respiratoires (Boulmene, Casablanca, Marrakech); **New Zealand:** MI Asher∗, University of Auckland (Auckland), R MacKay, Canterbury Health Laboratories (Nelson), C Moyes, Whakatane Hospital (Bay of Plenty), P Pattemore, University of Otago, Christchurch (Christchurch), N Pearce, London School of Hygiene and Tropical Medicine (Wellington); **Nigeria:** BO Onadeko, (Ibadan); **Panamá:** G Cukier∗, Hospital Materno Infantil Jose Domingo de Obaldia (David-Panamá); **Peru:** P Chiarella∗, Universidad Peruana de Ciencias Aplicadas [UPC] (Lima); **Philippines:** F Cua-Lim∗†, University of Santo Tomas (Metro Manila); **Poland:** A Brêborowicz, University of Medical Sciences (Poznan), G Lis∗, Jagiellonian University (Kraków); **Portugal:** R Câmara, Centro Hospitilar do Funchal (Funchal), ML Chiera, Hospital Pediátrico de Coimbra (Coimbra), JM Lopes dos Santos, Hospital Pedro Hispano (Porto), C Nunes, Center of Allergy and Immunology of Algarve (Portimao), J Rosado Pinto∗, Hospital da Luz (Lisbon); **Republic of Macedonia:** E Vlaski∗, University Children's Clinic (Skopje); **Samoa:** P Fuimaono V Pisi, (Apia); **SAR China:** G Wong, Prince of Wales Hospital (Hong Kong 13–14); **Singapore:** DY Goh, National University of Singapore (Singapore); **South Africa:** HJ Zar∗, University of Cape Town (Cape Town); **South Korea:** HB Lee∗, Hanyang University College of Medicine (Provincial Korea, Seoul); **Spain:** A Blanco-Quirós, Facultad de Medicina (Valladolid), RM Busquets, Universidad Autonoma de Barcelona (Barcelona), I Carvajal-Urueña, Centro de Salud de La Ería (Asturias), G García-Hernández, Hospital Universitario 12 de Octubre (Madrid), L García-Marcos∗, University of Murcia and Bio-health Research Institute of Murcia [IMIB] Arrixaca Research Institute (Cartagena), C González Díaz, Universidad del Pais Vasco [UPV/EHU] (Bilbao), A López-Silvarrey Varela, Fundacion Maria Jose Jove (A Coruña), M Morales-Suárez-Varela, Centro de Investigación Biomédica en Red de Epidemiología y Salud Pública [CIBERESP], Valencia University (Valencia), EG Pérez-Yarza, Universidad del Pais Vasco [UPV/EHU] (San Sebastián); **Sudan:** OA Musa, National Ribat University (Khartoum); **Sultanate of Oman:** O Al-Rawas∗, Sultan Qaboos University (Al-Khodh); **Syria:** S Mohammad∗, Tishreen University (Tartous), Y Mohammad, National Center for Research and Training in Chronic Respiratory Diseases - Tishreen University (Lattakia), K Tabbah, Aleppo University Hospital (Aleppo); **Taiwan:** JL Huang∗, Chang Gung University (Taipei), CC Kao, Kao-Chun-Chieh Clinic (Taoyuan); **Thailand:** M Trakultivakorn, Chiang Mai University (Chiang Mai), P Vichyanond∗, Mahidol University (Bangkok); **Tokelau:** T Iosefa∗, Ministry of Health (Tokelau); **United Kingdom:** M Burr†, Cardiff University Neuadd Meirionnydd (Wales), D Strachan, Population Health Research Institute, St George's, University of London (Surrey/Sussex); **Uruguay:** D Holgado∗, Hospital Pereira Rossell (Montevideo), MC Lapides, Hospital Paysandú (Paysandú); **USA:** HH Windom, Asthma and Allergy Research Center (Sarasota); **Venezuela:** O Aldrey∗, Jefe del Instituto (Caracas).

∗National Coordinators. †Deceased.

***Additional ISAAC National Coordinators:* Brazil:** D Solé, Universidade Federal de São Paulo; **Canada:** M Sears, McMaster University; **Chile:** V Aguirre, Hospital CRS El Pino; **Ecuador:** S Barba, AXXIS-Medical Centre, Sociedad Ecuatoriana de Alergologia, Inmunologia y Ciencias Afines [SEAICA]; **India:** J Shah, Jaslok Hospital & Research Centre; **Indonesia:** K Baratawidjaja, University of Indonesia; **Malaysia:** J de Bruyne, University of Malaya; **Samoa:** N Tuuau-Potoi, Ministry of Health, Samoa; **SAR China:** CK Lai, The Chinese University of Hong Kong; **Singapore:** BW Lee, National University of Singapore; **Sudan:** A El Sony, Epidemiological Laboratory (Epi-Lab) for Public Health, Research and Development; **United Kingdom, Channel Islands, Isle of Man:** R Anderson, Population Health Research Institute, St George's, University of London.
