# Supplementary material for: Comparison of individual-level and population-level risk factors for rhinoconjunctivitis, asthma, and eczema in the International Study of Asthma and Allergies in Childhood (ISAAC) Phase Three
Source: World Allergy Organ J. 2020 Jul 2;13(6):100123. doi: 10.1016/j.waojou.2020.100123 (PMC7334817; doi:10.1016/j.waojou.2020.100123)
Supplement: Multimedia component 1 [file mmc1.docx]

**SUPPORTING MATERIAL**

**Table S1: Risk factor definitions.**

**Table S2: Single outcome models of fully adjusted within school effects of exposures using the synthesis sample, stratified by country-level affluence. Mixed logistic regression models with random intercepts at the school, centre and country levels.**

**Table S3: Fully adjusted unimorbid two-way models split by country affluence. Mixed logistic regression models with random intercepts at the school, centre and country levels.**

**Figure S1. Synthesis sample data flowchart, age 6-7 years.**

**Figure S2. Synthesis sample data flowchart, age 13-14 years.**

**Figure S3. Triangular graphs showing unimorbid two-way comparisons of early life risk factor effects for 6-7-year-old children.**

**Figure S4. Triangular graphs showing unimorbid two-way comparisons of current risk factor effects for 6-7-year-old children.**

**Figure S5. Triangular graphs showing unimorbid two-way comparisons of current risk factor effects for 13-14-year-old adolescents.**

**Figure S6. Triangular graphs showing unimorbid two-way comparisons of effects of early life risk factors for 6-7-year-old children in affluent and non-affluent countries.**

**Figure S7. Triangular graphs showing unimorbid two-way comparisons of effects of current risk factors for 6-7-year-old children in affluent and non-affluent countries.**

**Figure S8. Triangular graphs showing unimorbid two-way comparisons of effects of current risk factors for 13-14-year-old adolescents in affluent and non-affluent countries.**

**Table S1: Risk factor definitions**

| **Risk Factors for ages 6-7** | **Question (asked to parent)** | **Positive Response** |
| --- | --- | --- |
| Low birthweight | What was the weight of your child when he / she was born? | Less than 2.5kg |
| Breastfed ever | Was your child breastfed? | Yes |
| Farm animals (prenatal) | Has the child’s mother had regular (at least once a week) contact with farm animals (e.g. cattle, pigs, goats, sheep or poultry) while being pregnant with this child? | Yes |
| Farm animals (1st year) | In your child’s first year of life, did he / she have regular (at least once a week) contact with farm animals (e.g. cattle, pigs, goats, sheep or poultry)? | Yes |
| Cat (1st year) | Did you have a cat in your home during the first year of your child’s life? | Yes |
| Dog (1^st^ year) | Did you have a dog in your home during the first year of your child’s life? | Yes |
| Paracetamol (1st year) | In the first 12 months of your child’s life, did you usually give paracetamol for fever? | Yes |
| Antibiotics (1st year) | In the first 12 months of your child’s life, did your child have any antibiotics? | Yes |
| 2 or more siblings | How many older and younger brothers and sisters does your child have? | Total of 2 or more |
| Heavy truck traffic (current) | How often do trucks pass through the street where you live, on weekdays? | Frequently or almost the whole day |
| Fast food (current) | In the past 12 months, how often, on average did your child eat fast food / burgers? | At least once a week |
| Television (current) | During a normal week, how many hours a day (24 hours) does your child watch television? | At least one hour per day |
| Paternal tobacco (current) | Does your child’s father (or male guardian) smoke cigarettes? | Yes |
| Maternal tobacco (current) | Does your child’s mother (or female guardian) smoke cigarettes? | Yes |
| Paracetamol (current) | In the past 12 months, how often, on average, have you given your child paracetamol? | At least once per month |
| Open fire cooking (current) | In your house, what fuels are usually used for cooking? Electricity, gas, open fires, other | Any that include open fires |
|  |  |  |
| **Risk Factors for ages 13-14** | **Question (asked to child)** | **Positive Response** |
| 2 or more siblings | How many older and younger brothers and sisters do you have? | Total of 2 or more |
| Heavy truck traffic (current) | How often do trucks pass through the street where you live, on weekdays? | Frequently or almost the whole day |
| Fast food (current) | In the past 12 months, how often, on average did you eat fast food / burgers? | At least once a week |
| Television (current) | During a normal week, how many hours a day (24 hours) do you watch television? | At least one hour per day |
| Paternal tobacco (current) | Does your father (or male guardian) smoke cigarettes? | Yes |
| Maternal tobacco (current) | Does your mother (or female guardian) smoke cigarettes? | Yes |
| Paracetamol (current) | In the past 12 months, how often, on average, have you taken paracetamol? | At least once per month |
| Open fire cooking (current) | In your house, what fuels are usually used for cooking? Electricity, gas, open fires, other | Any that include open fires |

**Table S2: Single outcome models of fully adjusted^A^ within school effects of exposures using the synthesis sample^B^, stratified by country-level affluence. Mixed logistic regression models with random intercepts at the school, centre and country levels.**

| Age | Exposure |  | Affluent Countries (n = 41,831) | | |  | Non-affluent Countries (n = 75,032) | | |
| --- | --- | --- | --- | --- | --- | --- | --- | --- | --- |
|  |  |  | Rhinoconjunctivitis OR (95% CI) | Asthma  OR (95% CI) | Eczema  OR (95% CI) |  | Rhinoconjunctivitis OR (95% CI) | Asthma  OR (95% CI) | Eczema  OR (95% CI) |
| 6-7 years | Low birthweight |  | 1.02 (0.89, 1.16) | 1.17 (1.03, 1.31) | 0.93 (0.80, 1.07) |  | 1.05 (0.95, 1.16) | 1.14 (1.03, 1.26) | 0.87 (0.77, 0.99) |
|  | Breastfed ever |  | 1.03 (0.96, 1.11) | 1.02 (0.96, 1.10) | 1.15 (1.06, 1.25) |  | 0.96 (0.89, 1.04) | 0.87 (0.81, 0.94) | 1.05 (0.96, 1.15) |
|  | Farm animals (prenatal) |  | 1.16 (0.98, 1.38) | 0.98 (0.83, 1.14) | 1.00 (0.83, 1.21) |  | 1.20 (1.07, 1.34) | 1.32 (1.18, 1.49) | 1.20 (1.06, 1.36) |
|  | Farm animals (1st year) |  | 0.88 (0.75, 1.03) | 0.94 (0.81, 1.08) | 0.95 (0.80, 1.13) |  | 1.19 (1.07, 1.33) | 1.02 (0.91, 1.13) | 1.25 (1.11, 1.40) |
|  | Cat (1st year) |  | 1.05 (0.95, 1.16) | 1.10 (1.01, 1.20) | 0.99 (0.90, 1.09) |  | 1.11 (1.01, 1.22) | 1.36 (1.24, 1.49) | 1.23 (1.11, 1.36) |
|  | Dog (1st year) |  | 1.05 (0.97, 1.14) | 0.98 (0.91, 1.06) | 0.98 (0.90, 1.07) |  | 1.07 (1.00, 1.15) | 1.06 (0.99, 1.14) | 1.10 (1.02, 1.18) |
|  | Paracetamol (1st year) |  | 1.39 (1.27, 1.52) | 1.41 (1.30, 1.53) | 1.28 (1.17, 1.41) |  | 1.40 (1.31, 1.50) | 1.30 (1.21, 1.39) | 1.31 (1.22, 1.41) |
|  | Antibiotics (1st year) |  | 1.60 (1.49, 1.72) | 1.70 (1.59, 1.82) | 1.43 (1.33, 1.54) |  | 1.56 (1.46, 1.66) | 1.62 (1.52, 1.73) | 1.36 (1.27, 1.45) |
|  | 2 or more siblings |  | 0.87 (0.81, 0.94) | 0.91 (0.85, 0.98) | 0.95 (0.88, 1.02) |  | 1.06 (1.00, 1.13) | 1.01 (0.95, 1.07) | 0.93 (0.87, 1.00) |
|  | Heavy truck traffic (current) |  | 1.16 (1.08, 1.24) | 1.16 (1.09, 1.24) | 1.08 (1.01, 1.17) |  | 1.17 (1.11, 1.24) | 1.20 (1.14, 1.27) | 1.14 (1.07, 1.21) |
|  | Fast food (current) |  | 0.99 (0.92, 1.06) | 1.07 (1.00, 1.14) | 1.05 (0.97, 1.13) |  | 0.98 (0.92, 1.05) | 1.09 (1.03, 1.16) | 0.96 (0.89, 1.03) |
|  | Television (current) |  | 0.88 (0.81, 0.95) | 1.05 (0.98, 1.14) | 0.92 (0.85, 1.00) |  | 1.00 (0.92, 1.08) | 1.05 (0.97, 1.14) | 1.01 (0.93, 1.11) |
|  | Paternal tobacco (current) |  | 1.04 (0.97, 1.11) | 1.11 (1.03, 1.18) | 1.06 (0.98, 1.14) |  | 1.10 (1.03, 1.17) | 1.10 (1.03, 1.17) | 1.03 (0.96, 1.11) |
|  | Maternal tobacco (current) |  | 1.06 (0.98, 1.15) | 1.20 (1.12, 1.29) | 1.01 (0.92, 1.10) |  | 1.03 (0.94, 1.13) | 1.22 (1.11, 1.33) | 1.12 (1.02, 1.24) |
|  | Paracetamol (current) |  | 2.19 (2.01, 2.40) | 2.36 (2.19, 2.56) | 1.65 (1.50, 1.81) |  | 1.94 (1.82, 2.07) | 1.90 (1.78, 2.02) | 1.35 (1.25, 1.46) |
|  | Open fire cooking (current) |  | 1.52 (1.05, 2.21) | 1.62 (1.17, 2.24) | 1.09 (0.72, 1.65) |  | 0.88 (0.71, 1.09) | 1.10 (0.92, 1.32) | 1.13 (0.94, 1.36) |
| Age | Exposure |  | Affluent Countries (n = 46,932) | | |  | Non-Affluent Countries (n = 177,504) | | |
|  |  |  | Rhinoconjunctivitis OR (95% CI) | Asthma  OR (95% CI) | Eczema  OR (95% CI) |  | Rhinoconjunctivitis OR (95% CI) | Asthma  OR (95% CI) | Eczema  OR (95% CI) |
| 13-14 years | 2 or more siblings |  | 1.00 (0.94, 1.05) | 1.01 (0.95, 1.07) | 1.06 (0.97, 1.16) |  | 1.05 (1.02, 1.09) | 1.03 (0.99, 1.07) | 1.08 (1.03, 1.14) |
|  | Heavy truck traffic (current) |  | 1.22 (1.15, 1.28) | 1.15 (1.08, 1.22) | 1.33 (1.22, 1.45) |  | 1.23 (1.20, 1.27) | 1.21 (1.17, 1.26) | 1.31 (1.25, 1.36) |
|  | Fast food (current) |  | 1.06 (1.00, 1.11) | 1.03 (0.97, 1.09) | 1.06 (0.97, 1.16) |  | 1.06 (1.02, 1.09) | 1.08 (1.04, 1.12) | 1.06 (1.02, 1.11) |
|  | Television (current) |  | 0.98 (0.90, 1.08) | 1.05 (0.95, 1.16) | 0.95 (0.83, 1.10) |  | 1.01 (0.97, 1.06) | 1.01 (0.95, 1.06) | 1.10 (1.03, 1.18) |
|  | Paternal tobacco (current) |  | 1.02 (0.97, 1.08) | 1.10 (1.03, 1.16) | 1.04 (0.95, 1.14) |  | 1.12 (1.09, 1.16) | 1.11 (1.07, 1.16) | 1.18 (1.13, 1.23) |
|  | Maternal tobacco (current) |  | 1.13 (1.06, 1.20) | 1.27 (1.20, 1.36) | 1.07 (0.97, 1.19) |  | 1.14 (1.09, 1.19) | 1.19 (1.14, 1.25) | 1.12 (1.06, 1.19) |
|  | Paracetamol (current) |  | 1.96 (1.85, 2.08) | 1.99 (1.87, 2.11) | 1.76 (1.61, 1.93) |  | 1.70 (1.65, 1.76) | 1.75 (1.68, 1.81) | 1.54 (1.48, 1.61) |
|  | Open fire cooking (current) |  | 0.82 (0.62, 1.07) | 1.06 (0.82, 1.37) | 1.63 (1.16, 2.29) |  | 1.20 (1.11, 1.30) | 1.21 (1.11, 1.33) | 1.46 (1.32, 1.63) |

^A^Adjusted for sex, mother's level of education and all other variables in the table for that age group.

^B^Synthesis sample contains individuals with data present for all 3 outcomes, sex, maternal education and all exposures of interest.

**Table S3: Fully adjusted^A^ unimorbid two-way models split by country affluence. Mixed logistic regression models with random intercepts at the school, centre and country levels.**

| Exposure of Interest | Asthma v Eczema,  fully adjusted models^A^ | | Rhinoconjunctivitis v Asthma,  fully adjusted models^A^ | | Eczema v Rhinoconjunctivitis,  fully adjusted models^A^ | |
| --- | --- | --- | --- | --- | --- | --- |
| Age 6-7 years | Affluent countries  n=5,172 | Non-affluent countries  n=6,647 | Affluent countries  n=5,189 | Non-affluent countries  n=7,039 | Affluent countries  n=4,347 | Non-affluent countries  n=6,260 |
|  |  |  |  |  |  |  |
|  | OR (95% CI) | OR (95% CI) | OR (95% CI) | OR (95% CI) | OR (95% CI) | OR (95% CI) |
| Low birthweight | 1.42 (1.10, 1.82) | 1.40 (1.13, 1.72) | 0.81 (0.63, 1.05) | 0.90 (0.74, 1.09) | 0.85 (0.63, 1.14) | 0.93 (0.75, 1.15) |
| Breastfed ever | 0.86 (0.75, 0.99) | 0.84 (0.72, 0.99) | 1.03 (0.89, 1.18) | 1.09 (0.94, 1.27) | 1.12 (0.96, 1.31) | 1.10 (0.93, 1.29) |
| Farm animals (prenatal) | 0.85 (0.63, 1.16) | 1.13 (0.90, 1.42) | 1.23 (0.90, 1.68) | 0.92 (0.74, 1.15) | 0.85 (0.60, 1.20) | 1.01 (0.80, 1.27) |
| Farm animals (1st year) | 1.08 (0.82, 1.43) | 0.82 (0.67, 1.02) | 1.00 (0.76, 1.33) | 1.21 (0.98, 1.48) | 1.02 (0.75, 1.40) | 0.99 (0.81, 1.23) |
| Cat (1st year) | 1.08 (0.92, 1.27) | 1.17 (0.98, 1.39) | 0.91 (0.75, 1.09) | 0.69 (0.57, 0.82) | 1.06 (0.87, 1.29) | 1.35 (1.12, 1.63) |
| Dog (1st year) | 1.02 (0.87, 1.18) | 0.89 (0.78, 1.01) | 1.09 (0.93, 1.29) | 1.00 (0.88, 1.14) | 0.93 (0.78, 1.11) | 1.12 (0.98, 1.27) |
| Paracetamol (1st year) | 1.15 (0.99, 1.35) | 0.98 (0.86, 1.11) | 1.04 (0.88, 1.23) | 1.05 (0.92, 1.20) | 0.88 (0.73, 1.05) | 0.97 (0.86, 1.11) |
| Antibiotics (1st year) | 1.26 (1.11, 1.43) | 1.27 (1.12, 1.43) | 0.87 (0.76, 1.00) | 0.90 (0.80, 1.02) | 0.93 (0.81, 1.08) | 0.84 (0.75, 0.95) |
| 2 or more siblings | 0.98 (0.86, 1.12) | 1.04 (0.93, 1.18) | 0.89 (0.77, 1.03) | 1.11 (0.99, 1.24) | 1.11 (0.96, 1.30) | 0.89 (0.79, 1.00) |
| Heavy truck traffic (current) | 1.03 (0.91, 1.17) | 1.11 (1.00, 1.24) | 0.91 (0.80, 1.05) | 0.98 (0.88, 1.09) | 1.04 (0.90, 1.20) | 0.89 (0.80, 1.00) |
| Fast food (current) | 0.99 (0.87, 1.13) | 1.20 (1.07, 1.36) | 0.93 (0.81, 1.07) | 0.87 (0.77, 0.97) | 1.07 (0.92, 1.25) | 0.96 (0.85, 1.09) |
| Television (current) | 1.19 (1.02, 1.38) | 1.06 (0.90, 1.24) | 0.83 (0.71, 0.97) | 0.88 (0.76, 1.02) | 1.09 (0.92, 1.28) | 1.13 (0.96, 1.33) |
| Paternal tobacco (current) | 1.02 (0.89, 1.17) | 1.01 (0.90, 1.15) | 0.93 (0.81, 1.07) | 1.00 (0.89, 1.13) | 1.04 (0.89, 1.21) | 0.96 (0.85, 1.09) |
| Maternal tobacco (current) | 1.28 (1.11, 1.48) | 1.00 (0.84, 1.19) | 0.81 (0.70, 0.95) | 0.84 (0.71, 1.00) | 0.93 (0.78, 1.10) | 1.22 (1.03, 1.45) |
| Paracetamol (current) | 1.58 (1.34, 1.87) | 1.39 (1.22, 1.58) | 0.94 (0.79, 1.12) | 1.00 (0.89, 1.13) | 0.67 (0.55, 0.82) | 0.67 (0.59, 0.77) |
| Open fire cooking (current) | 1.21 (0.62, 2.35) | 1.00 (0.73, 1.38) | 1.00 (0.50, 2.03) | 0.72 (0.49, 1.04) | 0.82 (0.37, 1.81) | 1.54 (1.04, 2.26) |
| Age 13-14 years | Affluent countries  n=4,774 | Non-affluent countries  n=15,915 | Affluent countries  n=7,941 | Non-affluent countries  n=25,502 | Affluent countries  n=5,557 | Non-affluent countries  n=21,405 |
|  |  |  |  |  |  |  |
|  | OR (95% CI) | OR (95% CI) | OR (95% CI) | OR (95% CI) | OR (95% CI) | OR (95% CI) |
| 2 or more siblings | 0.93 (0.80, 1.07) | 0.95 (0.87, 1.03) | 0.98 (0.89, 1.09) | 1.01 (0.95, 1.08) | 1.07 (0.93, 1.23) | 1.03 (0.95, 1.11) |
| Heavy truck traffic (current) | 0.90 (0.78, 1.04) | 0.93 (0.87, 1.01) | 1.08 (0.98, 1.19) | 1.01 (0.96, 1.08) | 1.07 (0.93, 1.23) | 1.08 (1.01, 1.15) |
| Fast food (current) | 0.92 (0.80, 1.06) | 1.03 (0.95, 1.11) | 1.01 (0.91, 1.11) | 1.02 (0.96, 1.08) | 1.07 (0.93, 1.23) | 0.99 (0.92, 1.06) |
| Television (current) | 1.08 (0.85, 1.38) | 0.96 (0.85, 1.08) | 0.92 (0.77, 1.08) | 0.95 (0.87, 1.04) | 1.03 (0.81, 1.29) | 1.05 (0.94, 1.17) |
| Paternal tobacco (current) | 1.06 (0.92, 1.24) | 0.93 (0.86, 1.01) | 0.90 (0.81, 1.00) | 1.03 (0.96, 1.09) | 1.04 (0.90, 1.21) | 1.04 (0.97, 1.12) |
| Maternal tobacco (current) | 1.30 (1.11, 1.53) | 1.09 (0.98, 1.21) | 0.83 (0.74, 0.93) | 0.92 (0.85, 1.00) | 0.92 (0.79, 1.09) | 0.99 (0.90, 1.09) |
| Paracetamol (current) | 1.29 (1.10, 1.51) | 1.15 (1.06, 1.24) | 0.97 (0.87, 1.08) | 0.98 (0.92, 1.05) | 0.79 (0.68, 0.92) | 0.87 (0.81, 0.94) |
| Open fire cooking (current) | 0.77 (0.41, 1.45) | 0.92 (0.76, 1.11) | 0.73 (0.45, 1.19) | 0.97 (0.84, 1.13) | 1.87 (0.97, 3.59) | 1.20 (1.01, 1.44) |

^A^Adjusted for sex, mother's level of education and all other variables in the table for that age group.

**Figure S1. Synthesis sample data flowchart, age 6-7 years**

**
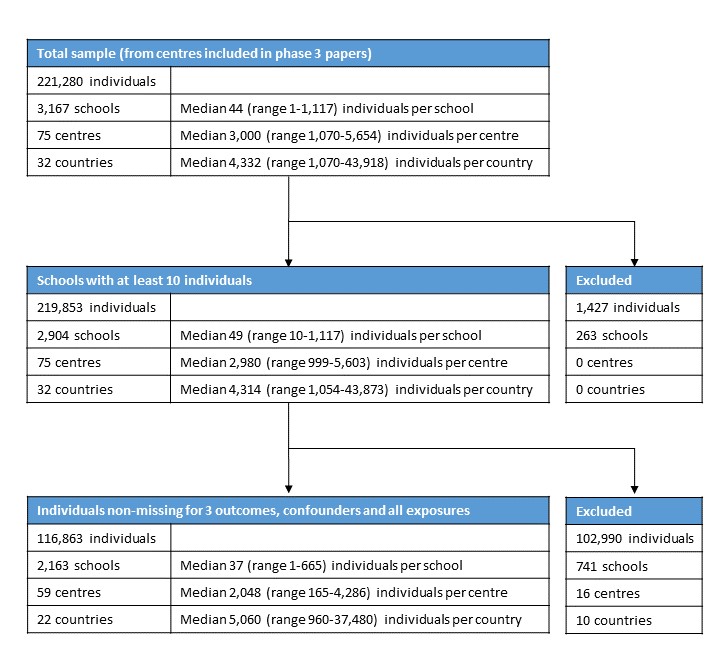
**

*Figure S1 shows the data flow through the exclusions to the final analysed sample for the 6-7 year-old children.*

**Figure S2. Synthesis sample data flowchart, age 13-14 years**


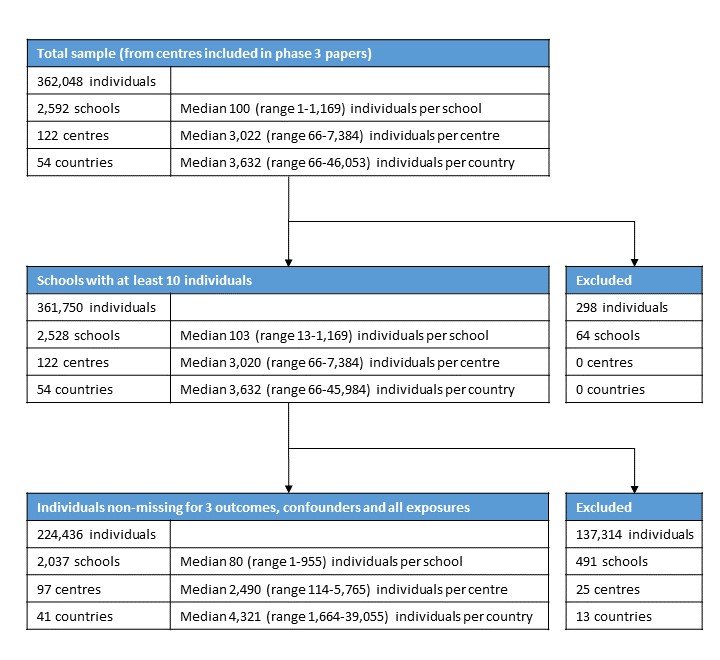


*Figure S2 shows the data flow through the exclusions to the final analysed sample for the 13-14 year-old adolescents.*

**Figure S3. Triangular graphs showing unimorbid two-way comparisons of early life risk factor effects for 6-7-year-old children.**

**
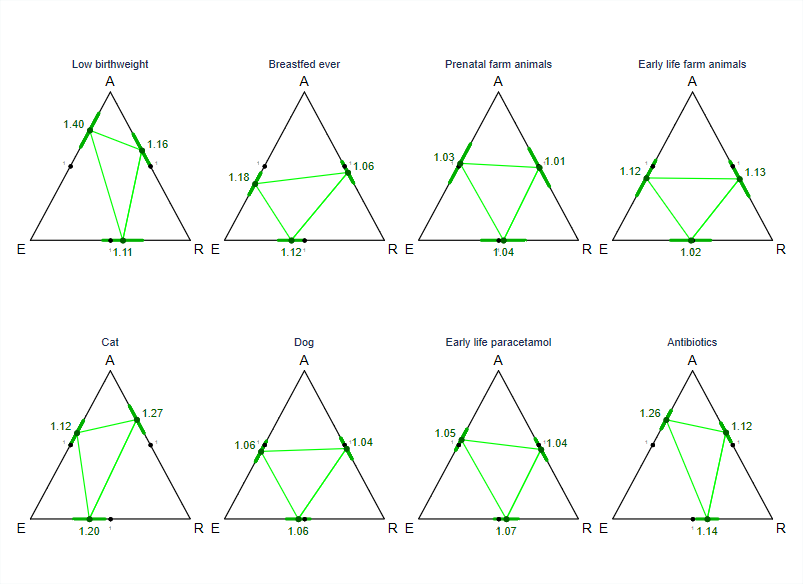
**

*Figure S3: Each triangular plot shows the OR (labelled) and 95% confidence interval for two-way associations between the risk factor and two of the three diseases Asthma (A), Eczema (E) and Rhinoconjunctivitis (R) using a sample of unimorbid individuals. The odds ratio is always over 1 and relates to the increased chance of an individual with that risk factor as having one disease over the other. It indicates a relative strength of association (one disease compared to another) rather than an absolute strength of association (a disease compared to no disease). An equilateral central triangle denotes a risk factor that has a similar strength of effect on all three diseases. The further from equilateral the triangle is, the more that risk factor effect differs in strength between diseases. Early life risk factors in 6-7 year-old children includes factors from the first year of the child’s life (except prenatal farm animals which is mother’s contact with farm animals during pregnancy with the child). The possible range of OR graphed is from 1 in the centre to 2 at either extreme, on the log scale.*

**Figure S4. Triangular graphs showing unimorbid two-way comparisons of current risk factor effects for 6-7-year-old children.**


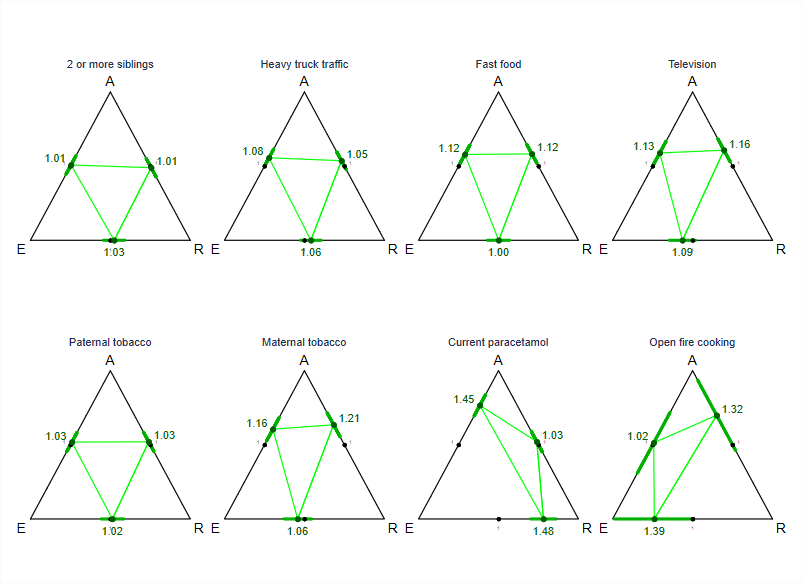


*Figure S4: Each triangular plot shows the OR (labelled) and 95% confidence interval for two-way associations between the risk factor and two of the three diseases Asthma (A), Eczema (E) and Rhinoconjunctivitis (R) using a sample of unimorbid individuals. The odds ratio is always over 1 and relates to the increased chance of an individual with that risk factor as having one disease over the other. It indicates a relative strength of association (one disease compared to another) rather than an absolute strength of association (a disease compared to no disease). An equilateral central triangle denotes a risk factor that has a similar strength of effect on all three diseases. The further from equilateral the triangle is, the more that risk factor effect differs in strength between diseases. Current risk factors in 6-7 year-old children includes factors from the previous 12 months. The possible range of OR graphed is from 1 in the centre to 2 at either extreme, on the log scale.*

**Figure S5. Triangular graphs showing unimorbid two-way comparisons of current risk factor effects for 13-14-year-old adolescents.**


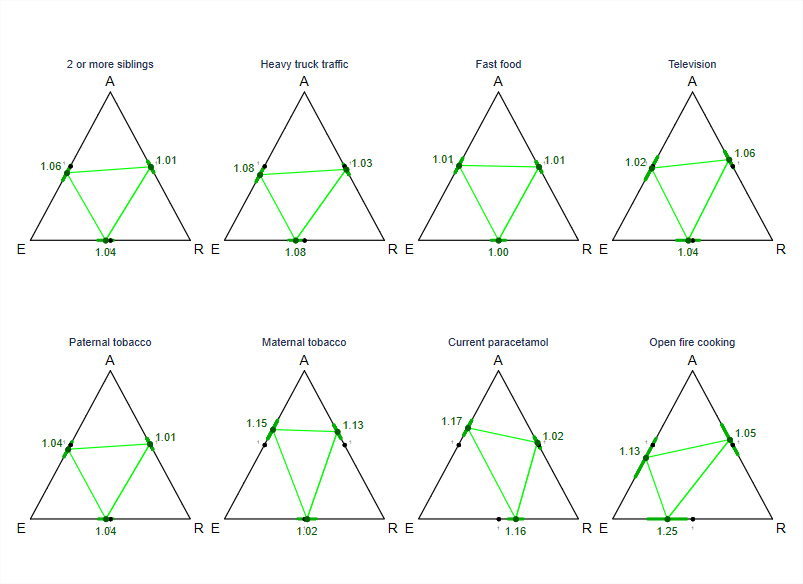


*Figure S5: Each triangular plot shows the OR (labelled) and 95% confidence interval for two-way associations between the risk factor and two of the three diseases Asthma (A), Eczema (E) and Rhinoconjunctivitis (R) using a sample of unimorbid individuals. The odds ratio is always over 1 and relates to the increased chance of an individual with that risk factor as having one disease over the other. It indicates a relative strength of association (one disease compared to another) rather than an absolute strength of association (a disease compared to no disease). An equilateral central triangle denotes a risk factor that has a similar strength of effect on all three diseases. The further from equilateral the triangle is, the more that risk factor effect differs in strength between diseases. Current risk factors in 13-14 year-old adolescents includes factors from the previous 12 months. The possible range of OR graphed is from 1 in the centre to 2 at either extreme, on the log scale.*

**Figure S6. Triangular graphs showing unimorbid two-way comparisons of effects of early life risk factors for 6-7-year-old children in affluent and non-affluent countries.**

**
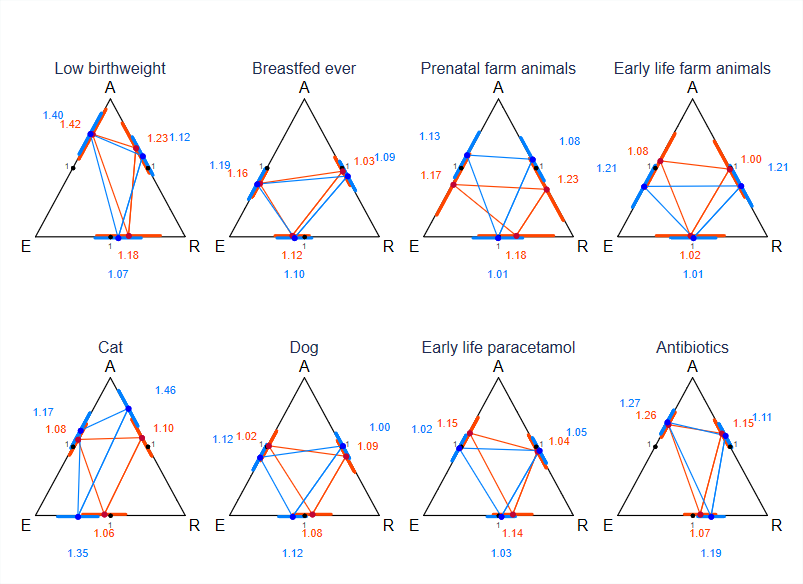
**

*Figure S6: Each triangular plot shows the OR (labelled) and 95% confidence interval for two-way associations between the risk factor and two of the three diseases Asthma (A), Eczema (E) and Rhinoconjunctivitis (R) using a sample of unimorbid individuals, stratified by affluent countries (red) and non-affluent countries (blue). The odds ratio is always over 1 and relates to the increased chance of an individual with that risk factor as having one disease over the other. It indicates a relative strength of association (one disease compared to another) rather than an absolute strength of association (a disease compared to no disease). An equilateral central triangle denotes a risk factor that has a similar strength of effect on all three diseases. The further from equilateral the triangle is, the more that risk factor effect differs in strength between diseases. Early life risk factors in 6-7 year-old children includes factors from the first year of the child’s life (except prenatal farm animals which is mother’s contact with farm animals during pregnancy with the child). The possible range of OR graphed is from 1 in the centre to 2 at either extreme, on the log scale.*

**Figure S7. Triangular graphs showing unimorbid two-way comparisons of effects of current risk factors for 6-7-year-old children in affluent and non-affluent countries.**

**
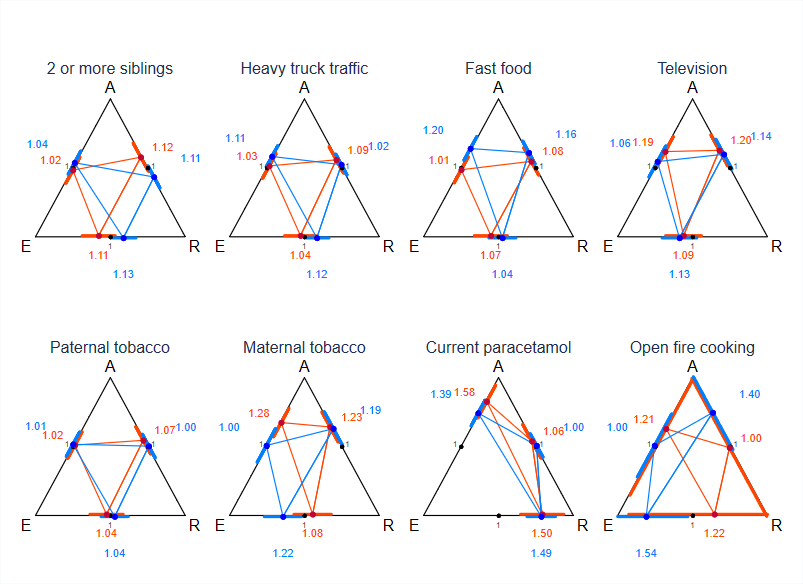
**

*Figure S7: Each triangular plot shows the OR (labelled) and 95% confidence interval for two-way associations between the risk factor and two of the three diseases Asthma (A), Eczema (E) and Rhinoconjunctivitis (R) using a sample of unimorbid individuals, stratified by affluent countries (red) and non-affluent countries (blue). The odds ratio is always over 1 and relates to the increased chance of an individual with that risk factor as having one disease over the other. It indicates a relative strength of association (one disease compared to another) rather than an absolute strength of association (a disease compared to no disease). An equilateral central triangle denotes a risk factor that has a similar strength of effect on all three diseases. The further from equilateral the triangle is, the more that risk factor effect differs in strength between diseases. Current risk factors in 6-7 year-old children includes factors from the previous 12 months. The possible range of OR graphed is from 1 in the centre to 2 at either extreme, on the log scale.*

**Figure S8. Triangular graphs showing unimorbid two-way comparisons of effects of current risk factors for 13-14-year-old adolescents in affluent and non-affluent countries.**

**
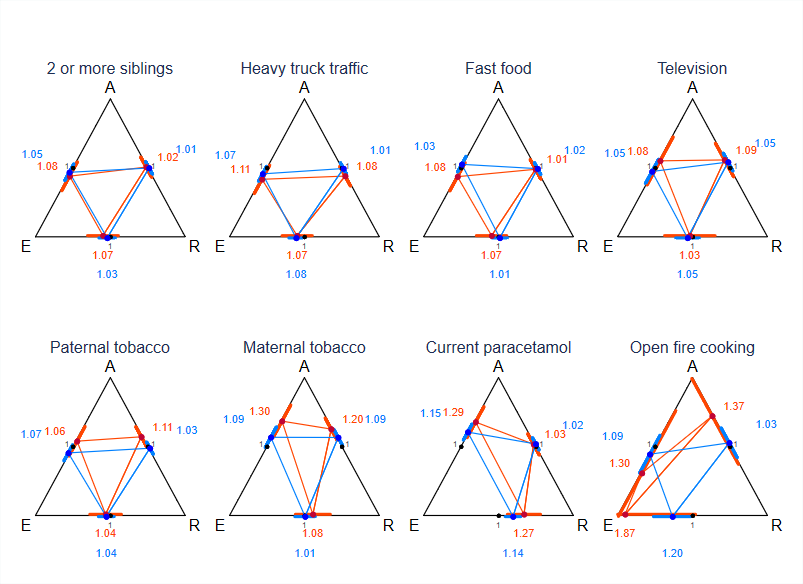
**

*Figure S8: Each triangular plot shows the OR (labelled) and 95% confidence interval for two-way associations between the risk factor and two of the three diseases Asthma (A), Eczema (E) and Rhinoconjunctivitis (R) using a sample of unimorbid individuals, stratified by affluent countries (red) and non-affluent countries (blue). The odds ratio is always over 1 and relates to the increased chance of an individual with that risk factor as having one disease over the other. It indicates a relative strength of association (one disease compared to another) rather than an absolute strength of association (a disease compared to no disease). An equilateral central triangle denotes a risk factor that has a similar strength of effect on all three diseases. The further from equilateral the triangle is, the more that risk factor effect differs in strength between diseases. Current risk factors in 13-14 year-old adolescents includes factors from the previous 12 months. The possible range of OR graphed is from 1 in the centre to 2 at either extreme, on the log scale.*
